# Muscle-level evaluation of the minimum muscle-stress-change model in human three-joint reaching using anatomically expanded arm models

**DOI:** 10.1007/s00422-026-01056-2

**Published:** 2026-08-22

**Authors:** Masazumi Katayama

**Affiliations:** Department of Human and Artificial Intelligent Systems, Graduate School of Engineering, University of Fukui, 3-9-1 Bunkyo, Fukui-shi, Fukui 910-8507 Japan

**Keywords:** Computational model, Reaching, Arm posture, Muscle tension, Muscle stress, Muscle recruitment

## Abstract

This study examined whether muscle-level optimization can account for both arm movements and muscle recruitment in planar three-joint reaching. To extend previous movement-level analyses based on simplified musculoskeletal representations, anatomically detailed three-joint arm models comprising 19–26 muscles with nonlinear, joint-angle-dependent moment arms were used. This extension enabled direct evaluation of whether muscle-level computational models can resolve highly redundant muscle-tension distributions. The reaching task was designed with eight directions at $\text {45}^\circ$ intervals in the horizontal plane, allowing evaluation with reduced directional bias. Several computational models were compared, with particular emphasis on the minimum muscle-stress-change (MSC) framework. The quadratic $\text {MSC}_2$, cubic $\text {MSC}_3$, and minimum muscle-tension-change (MTC) models reproduced the measured arm movements reasonably well; among the muscle-level models, $\text {MSC}_2$ provided the most accurate predictions, particularly for arm posture and the direction-dependent wrist-joint contribution. This level of accuracy justified evaluation of the predicted muscle-recruitment patterns. The MSC framework preferentially recruited muscles with larger physiological cross-sectional areas and avoided excessive loading of thinner muscles, whereas the MTC model was more strongly influenced by moment-arm values and sometimes assigned relatively large tensions to thinner muscles. The $\text {MSC}_3$ model recruited nearly all muscles in the 26-muscle expanded arm model but showed slightly lower movement-reproduction accuracy than the $\text {MSC}_2$ model. Thus, $\text {MSC}_2$ provided a balanced account of task-space behavior, joint-space coordination, and muscle recruitment. These findings extend muscle-stress-based optimization from movement reproduction to physiologically interpretable recruitment prediction and suggest that synergy-like cooperative patterns can emerge from anatomical constraints and stress-based optimization.

**Supplementary Information:**

The online version contains supplementary material available at 10.1007/s00422-026-01056-2.

## Introduction

The generation of human arm movements requires the nervous system to determine a spatial path for the fingertip, a time-varying arm configuration, and a feasible muscle-tension distribution across redundant muscles. Previous computational models have mainly focused on the trajectory and posture components of this problem, including the minimum hand-jerk model (Flash and Hogan 1985), the minimum angular-jerk (AJ) model (Hogan 1984; Nakano et al. 1999; Wada et al. 2001), and the minimum torque-change (TC) model (Uno et al. 1989; Nakano et al. 1999). Although these models have contributed substantially to understanding movement selection, they do not specify how the required joint torques are implemented by individual muscles. To examine muscle recruitment, the optimization criterion must be formulated at the muscle level. Models such as the minimum muscle-tension-change (MTC) model (Dornay et al. 1996), the minimum motor-command-change model (Kawato 1996), and the minimum muscle-stress-change (MSC) model (Katayama 2025) provide such a framework because they compute muscle tensions while satisfying musculoskeletal constraints. Katayama (2025) showed that the MSC model could reproduce fingertip trajectories and arm postures in human three-joint reaching with accuracy comparable to, or better than, that of the AJ and TC models. Previous studies of these computational models have often used simplified arm models, such as a two-joint, six-muscle model (e.g., Katayama and Kawato 1993) or the three-joint, eight-muscle model used by Katayama (2025). These reduced models are useful for evaluating whether a computational model can reproduce arm movements at the level of fingertip trajectories and arm postures. However, because they include only a minimal set of agonist–antagonist muscle pairs, they are insufficient for examining how redundancy among many muscles is resolved or for predicting physiologically meaningful muscle recruitment. Thus, Katayama (2025) tested whether MSC-based optimization could reproduce arm movements using a three-joint, eight-muscle model with constant moment arms, but did not thoroughly investigate whether the model could generate anatomically interpretable recruitment patterns.

To predict muscle recruitment during arm movements, the arm model must include a number of muscles comparable to that in the human musculoskeletal system. High muscular redundancy is particularly important because many muscles act around the same joint and multiple muscles with similar functional roles coexist. However, increasing only the number of muscles is not sufficient. The model must also represent anatomical differences among muscles, especially differences in physiological cross-sectional area (PCSA) and moment arm. Accurate PCSA and moment-arm values are therefore essential for evaluating muscle recruitment predicted by the MSC framework. PCSA values determine how muscle stresses are computed, whereas moment arms determine how muscle tensions generate joint torques. Because moment arms change with joint angle during movement, joint-angle-dependent moment arms are required for realistic evaluation of muscle recruitment. A more detailed musculoskeletal arm model is consequently needed to evaluate whether a computational principle can select muscle recruitment patterns that reflect the balance among muscles with different PCSAs and angle-dependent moment arms.

Accordingly, the present study evaluates the $\text {MSC}_2$, $\text {MSC}_3$, MTC, combined $\text {MTC}_s$, and minimum muscle-torque-change (MTRC) models using anatomically detailed three-joint arm models, with the AJ model used as a benchmark for evaluating the reproducibility of arm movements. Model performance was assessed at three levels: task space (fingertip trajectories), joint space (arm postures and the direction-dependent wrist-joint contribution, $C_w$), and the muscle level (muscle recruitment and sensitivity to PCSA and moment-arm values). The present study therefore extends the work of Katayama (2025) from movement-level reproduction with a three-joint, eight-muscle arm model to muscle-recruitment prediction with anatomically expanded arm models.

## Movement selection by computational models

### Muscle-level computational models

The models evaluated here were selected to clarify how muscle-level cost functions determine both arm movements and muscle recruitment. Following the two-stage optimization framework described by Katayama (2025), optimal solutions were obtained for each muscle-level computational model. Common implementation details are therefore summarized briefly, whereas the muscle-level cost functions and differences from the previous eight-muscle model are emphasized.

Prior isometric force-production studies indicate that muscle tensions inferred from EMG can be described by stress-based optimization criteria, particularly quadratic and cubic formulations (Crowninshield and Brand 1981; van Bolhuis and Gielen 1999; Gomi 2001). This evidence provides physiological motivation for evaluating the MSC framework as a rule for muscle recruitment in the present anatomically expanded arm models. The evaluation functions based on the MSC framework are as follows:

1 $$\begin{aligned} &  \text {First stage:}\nonumber \\ &  {E_{MSC_p}} = \sum _{i=1}^{m}{S_i}^p, ~~~~~ S_i = \frac{T_i}{PCSA_i}, \end{aligned}$$

2 $$\begin{aligned} &  s.t.\boldsymbol{\tau } = \textbf{A}(\boldsymbol{\theta })\textbf{T},~0 \le T_i \le PCSA_i \times 60\; \mathrm {[N/cm^2]}, \nonumber \\ &  \text {Second stage:}\nonumber \\ &  {C_{MSC_p}} = \frac{1}{2}\int _0^{t_f} \sum _{i=1}^m \left| \frac{d S_{i}}{dt} \right| ^p dt. \end{aligned}$$

Here, *m* is the number of muscles in each three-joint arm model; $S_i$ and $T_i$ denote the stress and tension of the *i*-th muscle at time *t*, respectively; $\boldsymbol{\tau }$ is the joint-torque vector; $\textbf{T}$ is the vector of muscle tensions; $\boldsymbol{\theta }$ is the vector of joint angles; $\textbf{A}$ is the moment-arm matrix that depends on $\boldsymbol{\theta }$; $PCSA_i$ is the physiological cross-sectional area of the *i*-th muscle; and $t_f$ is the movement duration from the initial to the final position. Muscle stress is defined as $S_i=T_i/PCSA_i$. $\text {MSC}_2$ and $\text {MSC}_3$ denote the cases p=2 and p=3, respectively. In the first stage of the two-stage optimization, $\textbf{T}$ was determined at each time step by minimizing $E_{MSC_p}$ subject to (i) the joint torque–muscle tension relationship $\boldsymbol{\tau }=\textbf{A}(\boldsymbol{\theta })\textbf{T}$ and (ii) non-negativity and upper bounds on muscle tension, with maximal voluntary contraction stress limited to approximately 60 $\mathrm {N/cm^2}$ (Ikai and Fukunaga 1968; Crowninshield 1978). At the beginning and end of the movement, the arm was stationary: $\dot{\boldsymbol{\theta }}(0)=\dot{\boldsymbol{\theta }}(t_f)=\boldsymbol{0}$ and $\ddot{\boldsymbol{\theta }}(0)=\ddot{\boldsymbol{\theta }}(t_f)=\boldsymbol{0}$. Accordingly, the joint torques at both endpoints were set to zero. In the second-stage optimization, the muscle stresses calculated from the muscle tensions determined at all time steps in the first-stage optimization were used to calculate $C_{MSC_p}$. Following the RCGA framework described by Katayama (2025), this calculation was repeated while varying the fingertip trajectories, *x* and *y*, and the wrist-joint angle trajectory, $\theta _3$, until $C_{MSC_p}$ was minimized. The optimal fingertip and joint-angle trajectories and the corresponding muscle-tension profiles were thereby obtained. No additional constraints were imposed in the second-stage optimization because the muscle tensions determined in the first stage already satisfied all prescribed constraints.

The minimum muscle-tension-change (MTC) model was also evaluated as a muscle-level model because it determines the muscle-tension vector directly (e.g., Dornay et al. 1996). Following the two-stage approach used for the MSC framework, the MTC model computed optimal fingertip trajectories, arm postures, and recruitment patterns by minimizing $E_{MTC}$ in the first stage and $C_{MTC}$ in the second stage:

3 $$\begin{aligned} &  \text {First stage:} \nonumber \\ &  {E_{MTC}} = \sum _{i=1}^{m} {T_i}^2, \end{aligned}$$

4 $$\begin{aligned} &  s.t.\boldsymbol{\tau } = \textbf{A}(\boldsymbol{\theta })\textbf{T},~~ 0 \le T_i \le PCSA_i \times 60\; \mathrm {[N/cm^2]}, \nonumber \\ &  \text {Second stage:} \nonumber \\ &  {C_{MTC}} = \frac{1}{2}\int _0^{t_f} \sum _{i=1}^m \left( \frac{d T_{i}}{dt} \right) ^2 dt. \end{aligned}$$

The MSC models were primarily compared with the MTC model, but two additional computational models were evaluated for reference. First, the combined model ($\text {MTC}_s$), which minimized $E_{MSC_2}$ in the first-stage optimization and $C_{MTC}$ in the second-stage optimization, was evaluated. Second, the minimum muscle-torque-change (MTRC) model was examined. In this model, the second-stage objective function, $C_{MTRC}$, was minimized using the muscle tensions obtained by minimizing $E_{MSC_2}$ in Eq. 1:

5 $$\begin{aligned} \text {Second stage:} \nonumber \\ C_{MTRC}= &  \frac{1}{2} \int _0^{t_f} \sum _{j=1}^{3} \sum _{i\in \Omega _j} \left( \frac{dQ_{j,i}}{dt} \right) ^2 dt, \qquad \nonumber \\ Q_{j,i}= &  a_{j,i}(\theta _j)T_i. \end{aligned}$$

Here, $Q_{j,i}$ denotes the torque produced by the *i*-th muscle about the *j*-th joint, and $\Omega _j$ denotes the set of muscles acting about that joint. A biarticular muscle belongs to both $\Omega _1$ and $\Omega _2$, whereas each monoarticular muscle belongs to only one of the three sets. Therefore, the total number of muscle-torque terms is m+α, where α is the number of biarticular muscles.

The minimum angular-jerk (AJ) model was used only as a benchmark because it has been shown to reproduce human arm movements well but does not determine muscle recruitment (e.g., Katayama 2025). Specifically, the AJ model generated the optimal arm movements by minimizing the objective function $C_{AJ}$, defined as follows:

6 $$\begin{aligned} {C_{AJ}} = \frac{1}{2}\int _0^{t_f} \sum _{j=1}^3 \left( \frac{d^3 \theta _j}{dt^3} \right) ^2 dt, \end{aligned}$$

where $\theta _j$ denotes the joint angle of the *j*-th joint.

Model predictions were obtained using the RCGA framework described by Katayama (2025), but the present implementation was adapted to the expanded 19–26-muscle arm models. In addition to the $\text {MSC}_2$ model, the muscle-level computational models $\text {MSC}_3$, MTC, $\text {MTC}_s$, and MTRC were implemented. Quadratic first-stage problems were solved by quadratic programming, whereas the cubic stress criterion was optimized using the C++ interface of the NLopt library (Johnson 2008). Each condition was computed three times from different initial populations; the resulting solutions showed no substantive differences.

### Anatomically expanded arm models

A central difference from Katayama (2025) is the musculoskeletal representation. The previous model used eight muscles with constant moment arms, which was sufficient for evaluating movement-level reproduction but insufficient for discussing how individual anatomical muscles are recruited. In the present study, the model was expanded to 19–26 muscles, and moment arms were defined as joint-angle-dependent functions. This extension allows evaluation of whether each computational model recruits muscles in a manner consistent with anatomical PCSA and moment-arm properties.

The planar three-joint arm dynamics were represented by

7 $$\begin{aligned} &  \boldsymbol{\tau }= \textbf{A}(\boldsymbol{\theta })\textbf{T},\end{aligned}$$

8 $$\begin{aligned} &  \textbf{M}(\boldsymbol{\theta }) \boldsymbol{\ddot{\theta }} + \textbf{H}(\boldsymbol{\theta },\boldsymbol{\dot{\theta }}) + \textbf{D}(\boldsymbol{\tau }) \boldsymbol{\dot{\theta }} = {\boldsymbol{\tau }}, \end{aligned}$$

where $\boldsymbol{\tau }$ is the three-dimensional joint-torque vector for the shoulder, elbow, and wrist, $\boldsymbol{\tau }= (\tau _1, \tau _2, \tau _3)^T$. This joint-torque vector is generated by the muscle-tension vector ${\textbf{T}}$ through the moment-arm matrix ${\textbf{A}}(\boldsymbol{\theta })$. The state vector $\boldsymbol{\theta }$ represents the shoulder, elbow, and wrist angles, and its first and second derivatives represent angular velocity and angular acceleration. The matrices and vectors $\textbf{M}(\boldsymbol{\theta })$, $\textbf{D}(\boldsymbol{\tau })$, and $\textbf{H}(\boldsymbol{\theta },\boldsymbol{\dot{\theta }})$ describe inertial, viscous, and velocity-dependent dynamic components, respectively. Unlike the previous eight-muscle formulation, ${\textbf{A}}(\boldsymbol{\theta })$ has three rows and *m* columns, where *m* is the number of muscles included in each expanded arm model.

The muscle-tension vector $\textbf{T}$ is defined as

9 $$\begin{aligned} \textbf{T} = \left( \begin{array}{cccccccccccc} T_{s_1}&\ldots&T_{{s_2}}&T_{{e_1}}&\ldots&T_{{e_2}}&T_{{d_1}}&\ldots&T_{{d_2}}&T_{{w_1}}&\ldots&T_{{w_2}} \end{array}\right) ^{T}, \end{aligned}$$

and the moment-arm matrix $\textbf{A}(\boldsymbol{\theta })$ is expressed as

10 $$\begin{aligned} \textbf{A}(\boldsymbol{\theta }) = \left( \begin{array}{cccccccccccc} a_{1,{s_1}}(\theta _1) & \ldots & a_{1,{s_2}}(\theta _1) & 0 & \ldots & 0 & a_{1,{d_1}}(\theta _1) & \ldots & a_{1,{d_2}}(\theta _1) & 0 & \ldots & 0 \\ 0 & \ldots & 0 & a_{2,{e_1}}(\theta _2) & \ldots & a_{2,{e_2}}(\theta _2) & a_{2,{d_1}}(\theta _2) & \ldots & a_{2,{d_2}}(\theta _2) & 0 & \ldots & 0 \\ 0 & \ldots & 0 & 0 & \ldots & 0 & 0 & \ldots & 0 & a_{3,{w_1}}(\theta _3) & \ldots & a_{3,{w_2}}(\theta _3) \end{array}\right) , \end{aligned}$$

where $a_{j,i}$ denotes the moment arm of the *i*-th muscle about the *j*-th joint and is a function of the corresponding joint angle. For S22 in Table 2, subscripts were assigned as follows: ${s_1} = 1$ and ${s_2} = 11$ for the shoulder; ${e_1} = 12$ and ${e_2} = 17$ for the elbow; ${d_1} = 18$ and ${d_2} = 20$ for biarticular muscles spanning the shoulder and elbow; and ${w_1} = 21$ and $w_2 = m = 26$ for the wrist. As listed in Table 2, S11, S12, S21, and S22 include 19, 21, 21, and 26 muscles, respectively.

The joint-viscosity matrix $\textbf{D}$ was defined as

11 $$\begin{aligned} \textbf{D}(\boldsymbol{\tau })= &  \left[ \begin{array}{ccc} D_{11}(\tau _1) & D_{12}(\tau _2) & 0 \\ D_{21}(\tau _2) & D_{22}(\tau _2) & 0 \\ 0 & 0 & D_{33}(\tau _3) \end{array} \right] . \end{aligned}$$

In the viscosity matrix, the diagonal elements ($D_{11}$, $D_{22}$, and $D_{33}$) describe viscosity at the shoulder, elbow, and wrist, whereas the off-diagonal shoulder-elbow terms represent inter-joint viscous coupling. The shoulder and elbow viscosity coefficients were derived from empirical measurements of human planar reaching (Gomi and Kawato 1995). The wrist coefficient $D_{33}$ was specified as one-half of the average shoulder-elbow baseline value. Although joint viscosity was fixed in Katayama (2025), it was modeled here as a function of joint torque, which changes during movement, based on the results reported by Gomi and Kawato (1995).

12 $$\begin{aligned} \begin{aligned} D_{11}(\tau _1)&= 0.63 + 0.095 |\tau _1|, \\ D_{21}(\tau _2)&= D_{12}(\tau _2) = 0.175 + 0.0375 |\tau _2|, \\ D_{22}(\tau _2)&= 0.76 + 0.185 |\tau _2|, \\ D_{33}(\tau _3)&= 0.348 + 0.140 |\tau _3|. \end{aligned} \end{aligned}$$

Here, viscosity values are expressed in $\mathrm {N\,m\,s/rad}$.

**Fig. 1:**
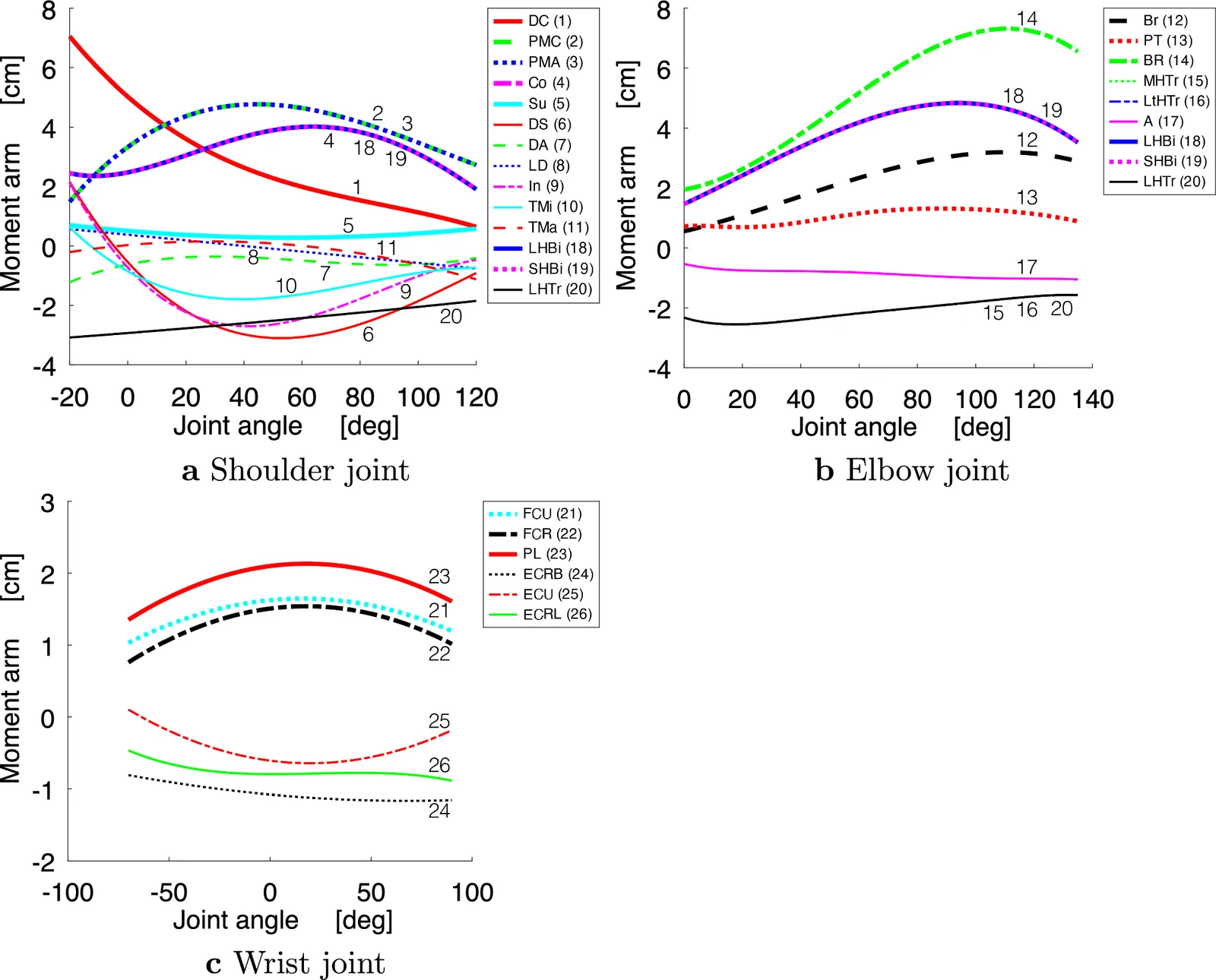
Moment-arm functions for the anatomical muscles in the three-joint arm models. Positive and negative moment arms indicate flexion- and extension-producing actions, respectively. Zero degrees correspond to a rightward upper arm, a fully extended elbow, and a hand aligned with the forearm. Muscle indices correspond to Table 2

For the muscle-level computational models, the key anatomical extension was the use of joint-angle-dependent moment arms. Based on measurements reported by Kuechle et al. (1997), Pigeon et al. (1996), and Amis et al. (1979), each moment arm was approximated as a polynomial function of joint angle (Fig. 1 and Table 5). This extension is particularly important for shoulder muscles, whose moment arms change substantially with joint angle. Because the MSC framework also requires PCSAs, model performance and recruitment were evaluated using the three PCSA sets listed in Table 2. In PCSA1, monoarticular shoulder-muscle PCSAs were determined from Meek et al. (1990), and the remaining PCSAs were determined from An et al. (1981). In PCSA2, monoarticular shoulder-muscle PCSAs were determined from Meek et al. (1990), and the remaining PCSAs were determined from Amis et al. (1979). In PCSA3, all PCSA values were determined from Holzbaur et al. (2005). The muscle abbreviations are as follows: (1) DC, deltoid (clavicular part); (2) PMC, pectoralis major (clavicular part); (3) PMA, pectoralis major (abdominal part); (4) Co, coracobrachialis; (5) Su, subscapularis; (6) DS, deltoid (scapular part); (7) DA, deltoid (acromial part); (8) LD, latissimus dorsi; (9) In, infraspinatus; (10) TMi, teres minor; (11) TMa, teres major; (12) Br, brachialis; (13) PT, pronator teres; (14) BR, brachioradialis; (15) MHTr, triceps brachii (medial head); (16) LtHTr, triceps brachii (lateral head); (17) A, anconeus; (18) LHBi, biceps brachii (long head); (19) SHBi, biceps brachii (short head); (20) LHTr, triceps brachii (long head); (21) FCU, flexor carpi ulnaris; (22) FCR, flexor carpi radialis; (23) PL, palmaris longus; (24) ECRB, extensor carpi radialis brevis; (25) ECU, extensor carpi ulnaris; and (26) ECRL, extensor carpi radialis longus. The muscle-set labels follow the convention of the earlier study, but their anatomical contents were redefined for the present multi-muscle model. S11 and S12 were based on Ito and Takano (2012), whereas S21 and S22 were based on Nakamura and Saito (1992); supplementary muscles were included only in S12 and S22. Participant-specific physical parameters used in the dynamics calculation of Eq. 8 were taken from the estimation procedure described in Katayama (2025) and are listed in Table 1.

**Table 1:** Participant-specific physical parameters used in the dynamics calculation. Values are reported as mean ± standard deviation. For segment *k*, $L_k$, $L_{gk}$, $M_k$, and $I_k$ denote segment length, center-of-mass distance, mass, and moment of inertia, respectively

$L_1$ (m)	$L_2$ (m)	$L_3$ (m)	$L_{g1}$ (m)	$L_{g2}$ (m)	$L_{g3}$ (m)
0.284 ± 0.020	0.245 ± 0.014	0.188 ± 0.019	0.112 ± 0.008	0.0909 ± 0.0043	0.0667 ± 0.0071
$M_1$ [kg]	$M_2$ [kg]	$M_3$ [kg]	$I_1$ [kg $\text {m}^2$]	$I_2$ [kg $\text {m}^2$]	$I_3$ [kg $\text {m}^2$]
1.654 ± 0.375	0.831 ± 0.130	0.455 ± 0.088	0.0383 ± 0.0124	0.0126 ± 0.0030	0.0039 ± 0.0014

**Fig. 2:**
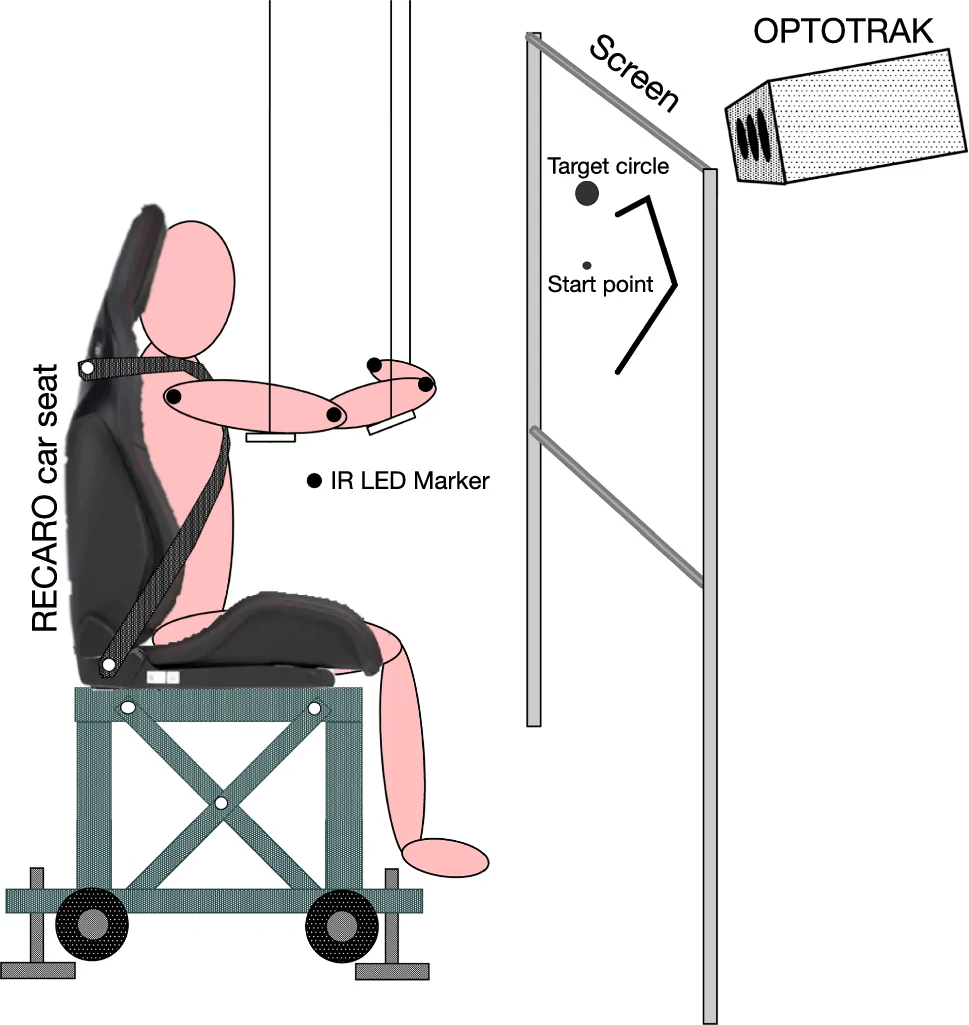
Planar-reaching apparatus used for the present experiment, based on the setup described by Katayama (2025). The display provided the start position, target circle, and online arm-configuration feedback, while ceiling suspension reduced gravitational loading of the arm

**Fig. 3:**
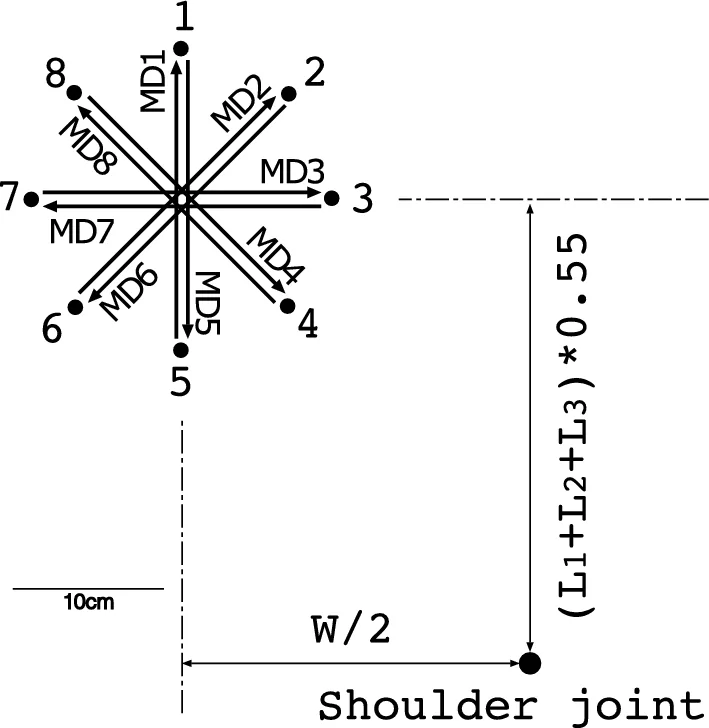
Eight-direction target arrangement defined in the horizontal reaching coordinate system. Target positions were defined at $\text {45}^\circ$ intervals around the start position. Because the target coordinates varied among participants according to their body dimensions, numerical coordinate axes are not shown; the scale bar represents 10 cm. *W*/2 denotes half the shoulder width, and $L_1$, $L_2$, and $L_3$ denote the upper-arm, forearm, and hand lengths, respectively

**Table 2:** Anatomical muscle sets and PCSA values used in the expanded arm models. Muscle types are abbreviated as SF, SE, EF, EE, DF, DE, WF, and WE for shoulder flexors, shoulder extensors, elbow flexors, elbow extensors, biarticular flexors, biarticular extensors, wrist flexors, and wrist extensors, respectively. Triangles indicate supplementary muscles in S12 and S22; arrows indicate that the PCSA value is inherited from the preceding column

Muscle	Muscle	Type	Muscle selection				PCSA [$\text {cm}^2$]		
#			S11	S12	S21	S22	PCSA1	PCSA2	PCSA3
*Monoarticular muscles of the shoulder joint*									
1	DC	SF	◯	◯	◯	◯	4.52	←	8.2
2	PMC	SF	◯	◯	◯	◯	5.16	←	2.6
3	PMA	SF	◯	◯	◯	◯	3.87	←	3.7
4	Co	SF	◯	◯	◯	◯	1.29	←	1.7
5	Su	SF			◯	◯	9.68	←	9.8
6	DS	SE	◯	◯	◯	◯	3.87	←	1.9
7	DA	SE			◯	◯	13.55	←	8.2
8	LD	SE		▵		▵	12.90	←	7.6
9	In	SE			◯	◯	5.81	←	8.6
10	TMi	SE			◯	◯	2.58	←	2.5
11	TMa	SE				▵	5.81	←	3.0
*Monoarticular muscles of the elbow joint*									
12	Br	EF	◯	◯	◯	◯	7.0	9.49	7.1
13	PT	EF	◯	◯		▵	3.4	4.37	4.0
14	BR	EF		▵		▵	1.5	3.22	1.9
15	MHTr	EE	◯	◯	◯	◯	6.1	14.46	4.5
16	LtHTr	EE	◯	◯	◯	◯	6.0	15.42	4.5
17	A	EE	◯	◯		▵	2.5	2.00	2.5
*Biarticular muscles of the shoulder and elbow joints*									
18	LHBi	DF	◯	◯	◯	◯	2.5	4.13	4.5
19	SHBi	DF	◯	◯	◯	◯	2.1	3.96	3.1
20	LHTr	DE	◯	◯	◯	◯	6.7	20.44	5.7
*Monoarticular muscles of the wrist joint*									
21	FCU	WF	◯	◯	◯	◯	3.2	5.97	2.9
22	FCR	WF	◯	◯	◯	◯	2.0	2.94	1.6
23	PL	WF	◯	◯	◯	◯	0.9	0.70	0.6
24	ECRB	WE	◯	◯	◯	◯	2.9	4.72	2.2
25	ECU	WE	◯	◯	◯	◯	3.4	4.72	2.1
26	ECRL	WE	◯	◯	◯	◯	2.4	3.30	2.2

**Table 3:** Observed movement durations for each reaching direction. Values are presented as mean ± standard error of the mean in seconds

MD1	MD2	MD3	MD4
0.538 ± 0.065	0.533 ± 0.060	0.637 ± 0.070	0.645 ± 0.074

MD5	MD6	MD7	MD8
0.527 ± 0.071	0.569 ± 0.073	0.642 ± 0.073	0.663 ± 0.087

## Measurement of three-joint reaching movements

Nine healthy right-handed adults participated in the experiment (age: 22.1 ± 1.1 years; height: 1.71 ± 0.08 m; two females). All participants reported normal or corrected-to-normal vision and were not informed of the detailed hypothesis before the experiment. Written informed consent was obtained from each participant, and participants received book coupons after participation. The study protocol was approved by the Human Research Ethics Committee of the Division of Human and Artificial Intelligent Systems at University of Fukui (#H181201).

The experimental apparatus and basic recording procedure were the same as those described in Katayama (2025), whereas the present analysis used an eight-direction target arrangement designed to evaluate movement and muscle recruitment without strong directional bias. Briefly, as illustrated in Fig. 2, participants were seated and stabilized with a four-point harness, and the hand, forearm, and upper arm were partially suspended from the ceiling to reduce gravitational loading during horizontal reaching. Four infrared markers were attached to the shoulder, elbow, wrist, and fingertip, and their three-dimensional positions were recorded at 200 Hz using an OPTOTRAK3020 system. All reaching movements were performed in a horizontal plane containing the shoulder, elbow, wrist, and fingertip. Thus, the upper arm, forearm, and hand moved within the horizontal reaching plane throughout each movement. The measured arm configuration was displayed in real time as a stick figure on a screen positioned in front of the participant and parallel to the participant’s coronal plane. The target positions shown in Fig. 3 were predefined in the horizontal reaching coordinate system but were not physically placed in the reaching plane. Instead, the start point and the target circle for each movement were displayed on the screen. Participants performed the reaching movements while viewing the real-time stick figure and the displayed target. This arrangement prevented the target display from being obscured by the participant’s moving arm.

Before data recording, participants practiced the task to become familiar with the apparatus and to perform relaxed reaching movements, using the same procedure as Katayama (2025). In the present experiment, targets were arranged in eight directions at $\text {45}^\circ$ intervals around the start position, as illustrated in Fig. 3. The movement distance was 20 cm so that all participants could perform all eight directions within the reachable workspace. Fifteen trials were recorded for each direction, and the order of directions was randomized. In each trial, participants briefly shook the hand near the start position after the first auditory cue, reached to the target after the second cue, and maintained the final posture until the final cue.

*Data analysis.* Marker trajectories were low-pass filtered in both the forward and reverse directions with a cutoff frequency of 10 Hz. For the fingertip position (*x*,  *y*), the apparent planar curvature, *C*, was calculated as follows: $C = |\dot{x} \ddot{y} - \ddot{x} \dot{y}| / (\dot{x}^2 + \dot{y}^2)^{3 \over 2}$. The calculated apparent curvature became extremely large near movement onset and offset, where tangential velocity was low, and its temporal profile clearly differed from that of tangential velocity. Movement onset was defined as the first point at which *C* exceeded 0.5$\,\textrm{mm}^{-1}$ when searching backward from the midpoint of the movement, following the method of Katayama (2025). Movement offset was defined similarly by searching forward from the midpoint. The term “apparent planar curvature” is used because the calculated value is sensitive to small positional fluctuations when tangential velocity is low and does not imply that the physical fingertip trajectory had such a large curvature near movement onset or offset. For each movement direction and participant, RMS errors between the measured and model-predicted movements were computed separately for the planar fingertip trajectory and the three joint-angle trajectories. Multiple comparisons among models were performed using the Tukey–Kramer method.

The characteristics of arm posture during reaching movements are particularly evident in wrist-joint rotation. In this study, these characteristics were evaluated using the ratio of the wrist-joint rotation angle to the total rotation angle of the three joints during movement. The contribution rate of the wrist joint, $C_w$, was defined as follows:

13 $$\begin{aligned} {C_w}= &  \frac{\Delta \theta _3}{\Delta \theta _1 + \Delta \theta _2 + \Delta \theta _3}, \nonumber \\ \Delta \theta _n= &  {\sum ^{N}_{t=1} |{\dot{\theta }_n(t)}| \Delta t}, \end{aligned}$$

where n=1,2, and 3 correspond to the shoulder, elbow, and wrist joints, respectively, and *N* is the number of time samples in each trial. Thus, $C_w$ reflects the relative contribution of the wrist throughout the movement, rather than only the final arm posture at the endpoint.

**Fig. 4:**
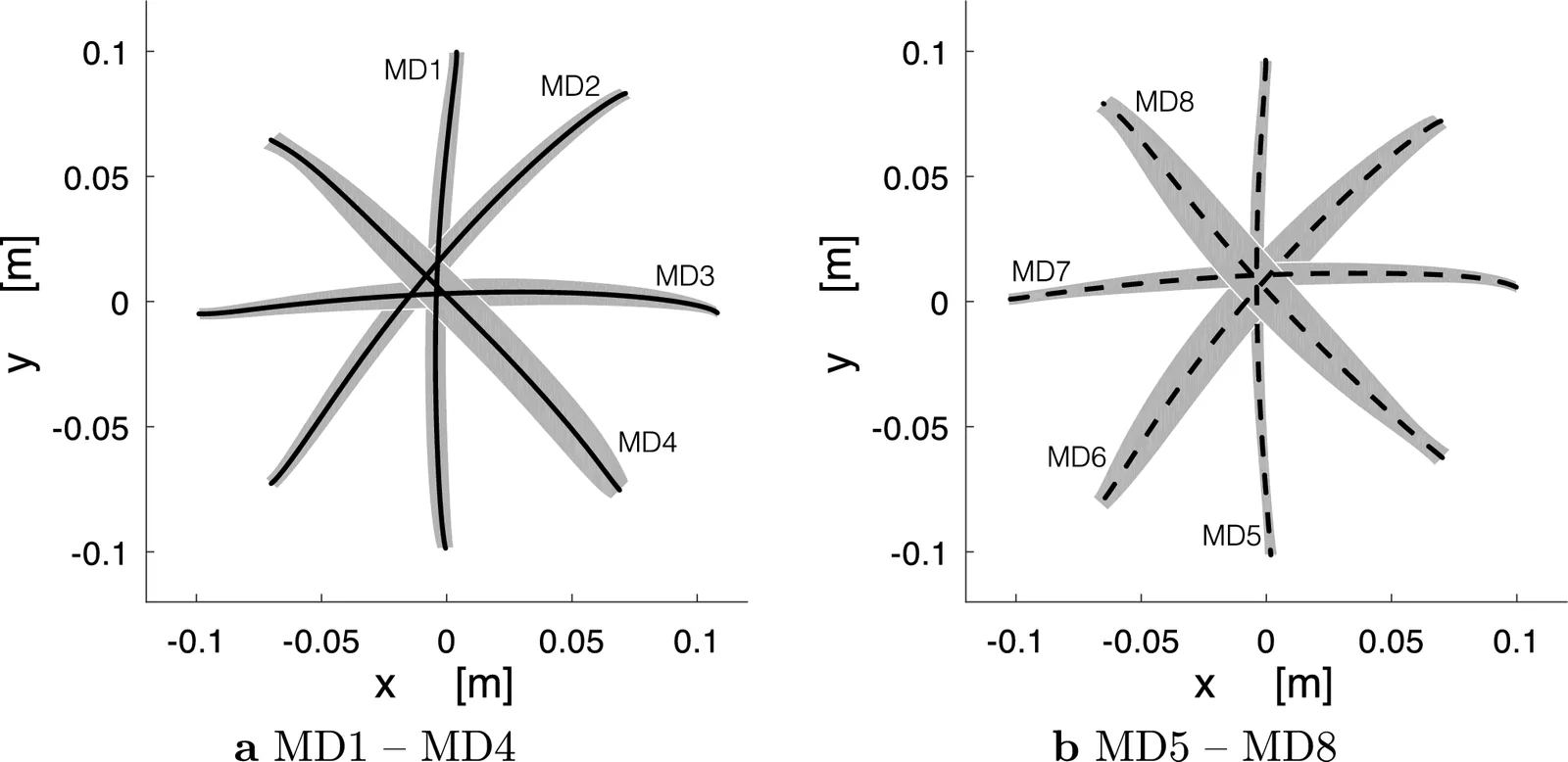
Mean fingertip paths across all participants. Solid lines represent the reaching movements for MD1–MD4, and dashed lines represent those for MD5–MD8. Because target locations varied across participants, the origin was placed at the center of each participant’s movement range. The shaded area indicates the standard error of the mean in the direction perpendicular to the line connecting the start and end points

**Fig. 5:**
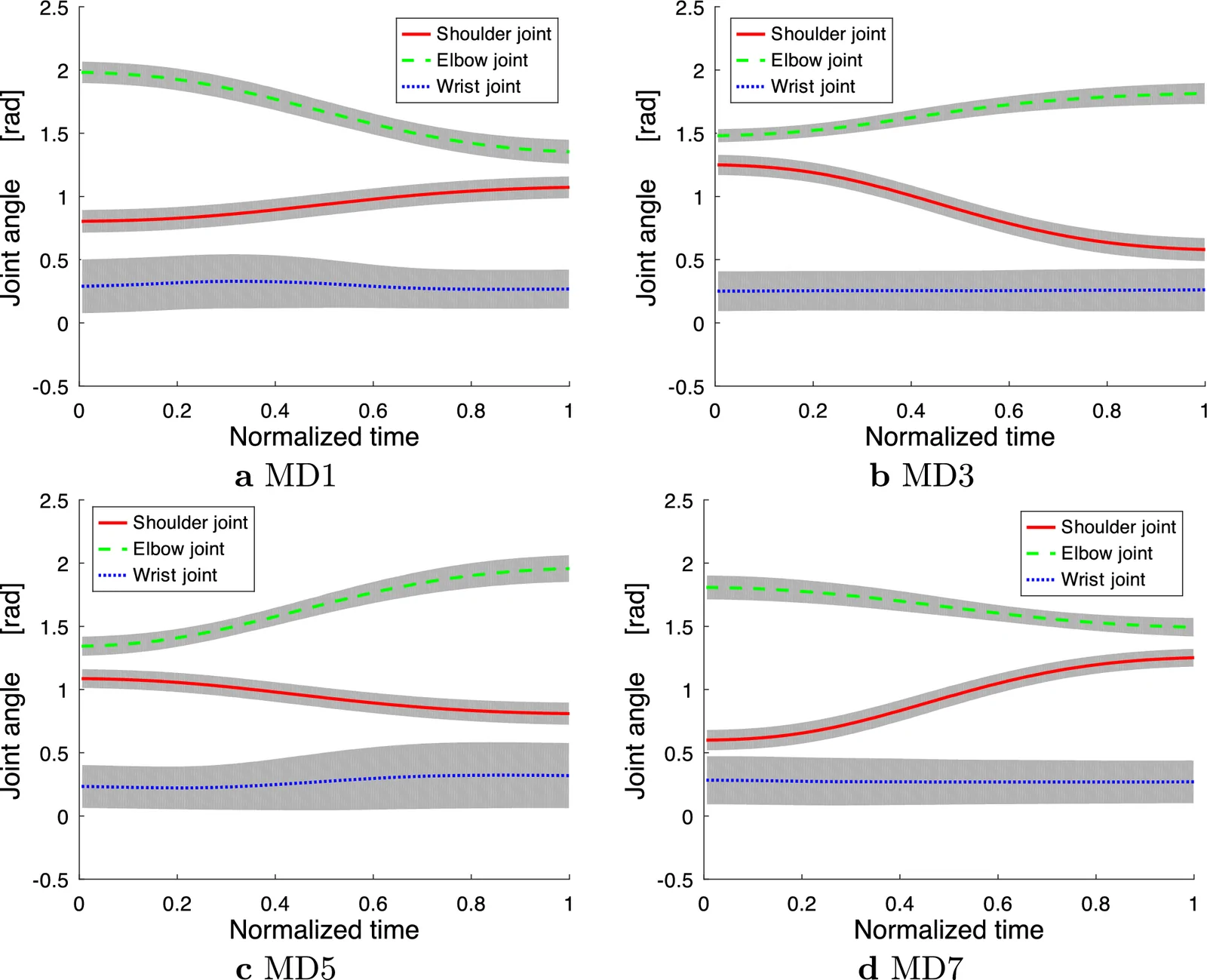
Mean joint angles across all participants. Time was normalized to the movement time of each trial. Joint angles of $\text {0}^\circ$ at the shoulder, elbow, and wrist indicate that the upper arm points to the right, the upper arm and forearm are aligned (i.e., the elbow is fully extended), and the forearm and hand are aligned, respectively. The shaded area indicates the standard error of the mean at each time point

**Fig. 6:**
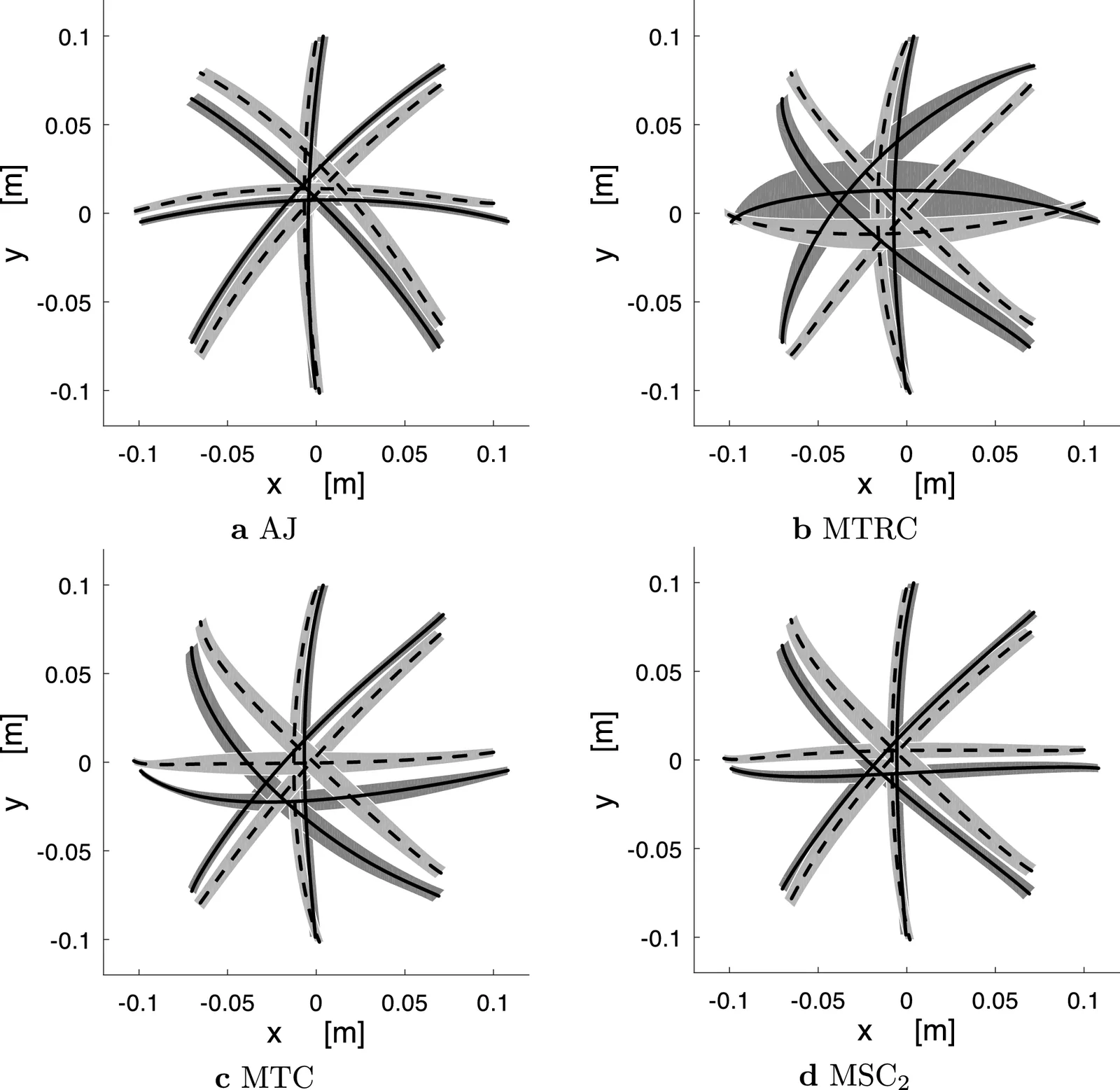
Fingertip trajectories generated by selected models. Solid traces show MD1–MD4, and dashed traces show MD5–MD8. Coordinates were centered on each participant’s movement range; shading represents the SEM perpendicular to the start-target axis. (Muscle selection: S22. PCSA: PCSA1)

**Fig. 7:**
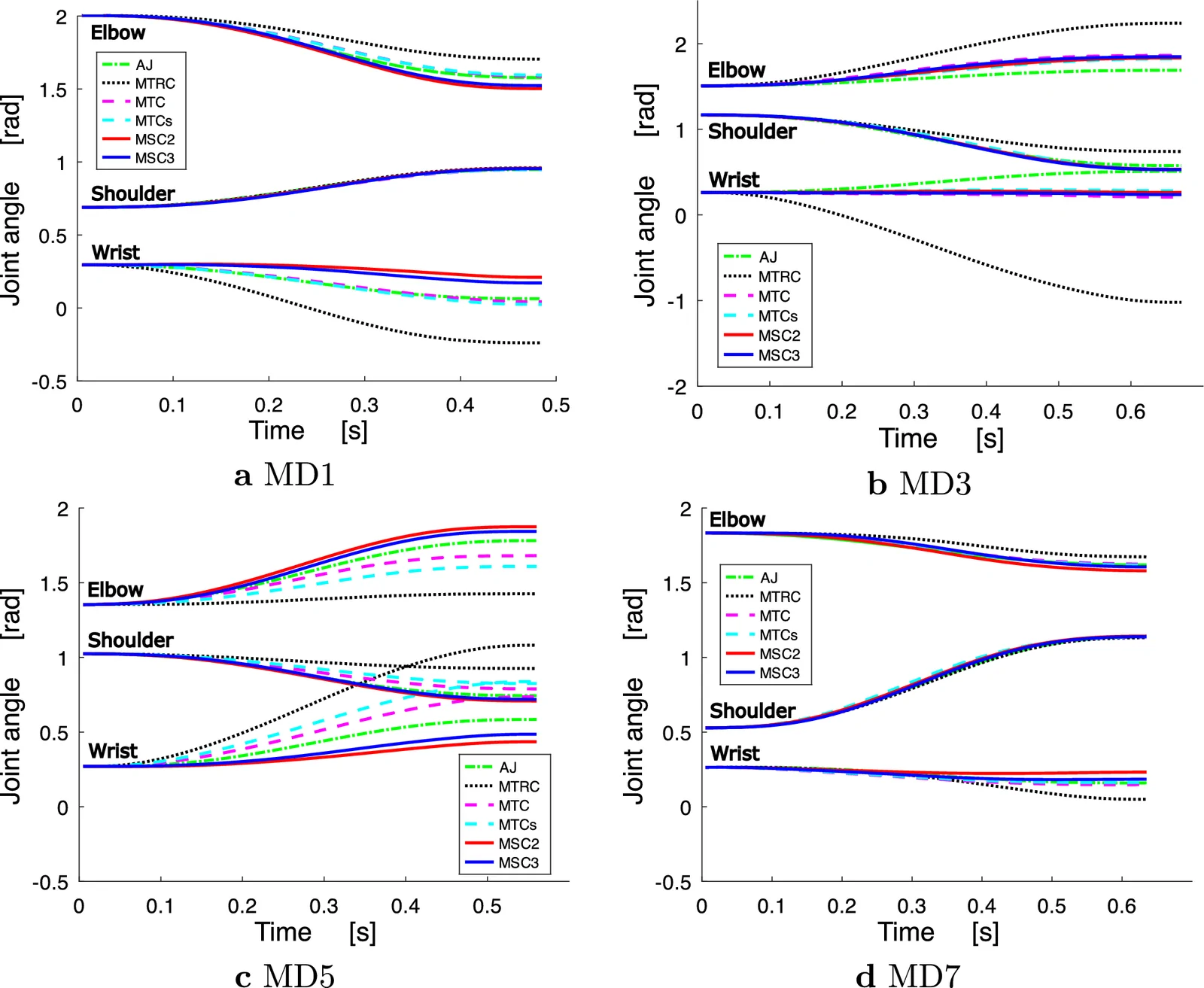
Optimal joint angles predicted by each computational model. Joint angles of $\text {0}^\circ$ at the shoulder, elbow, and wrist indicate that the upper arm points to the right, the upper arm and forearm are aligned (i.e., the elbow is fully extended), and the forearm and hand are aligned, respectively. (Muscle selection: S22. PCSA: PCSA1)

## Results

### Measured and optimal arm movements

As shown in Table 3, the measured movement times were close to the movement time specified during the practice session, although they were slightly longer in a few movement directions. Because the movement distance was approximately 20 cm, the measured fingertip paths were mostly straight (Fig. 4), but slight curvature was observed in some directions. Inter-participant variability in fingertip paths was greater for MD4, MD6, and MD8 than for the other directions. The measured arm postures also showed clear directional dependence (Fig. 5): in some directions, such as MD1 and MD5, the wrist rotated substantially, whereas in others, such as MD3 and MD7, wrist rotation was minimal. Although the initial posture differed across participants because of differences in link lengths, the temporal profiles of the joint angles were broadly similar.

Figure 6 shows the optimal fingertip paths generated by four selected computational models. Across all evaluated models, the AJ, $\text {MSC}_2$, and $\text {MSC}_3$ models produced less curved trajectories and smaller inter-participant variability than the other models. In MD1, MD5, and MD6, differences among the models were small. In the remaining directions, however, differences in fingertip paths were observed among the computational models, with the MTRC model in particular generating markedly curved fingertip paths. The MTC model also produced somewhat greater curvature than the AJ and $\text {MSC}_2$ models, especially in MD3 and MD4. In MD3, the fingertip path predicted by MTC curved in the opposite direction from those predicted by the AJ and MTRC models. In addition, the AJ model produced curvature opposite to that of the other models in MD4 and MD8, whereas the MTRC model produced curvature opposite to that of the AJ model in MD7. Figure 7 shows the optimal arm postures predicted by each model. The $\text {MSC}_2$ and $\text {MSC}_3$ models yielded smaller wrist rotations than the other models in all movement directions, with the smallest rotations observed for $\text {MSC}_2$. By contrast, the other models predicted larger wrist rotations during movement, and the MTRC model produced the largest wrist rotation overall.

**Fig. 8:**
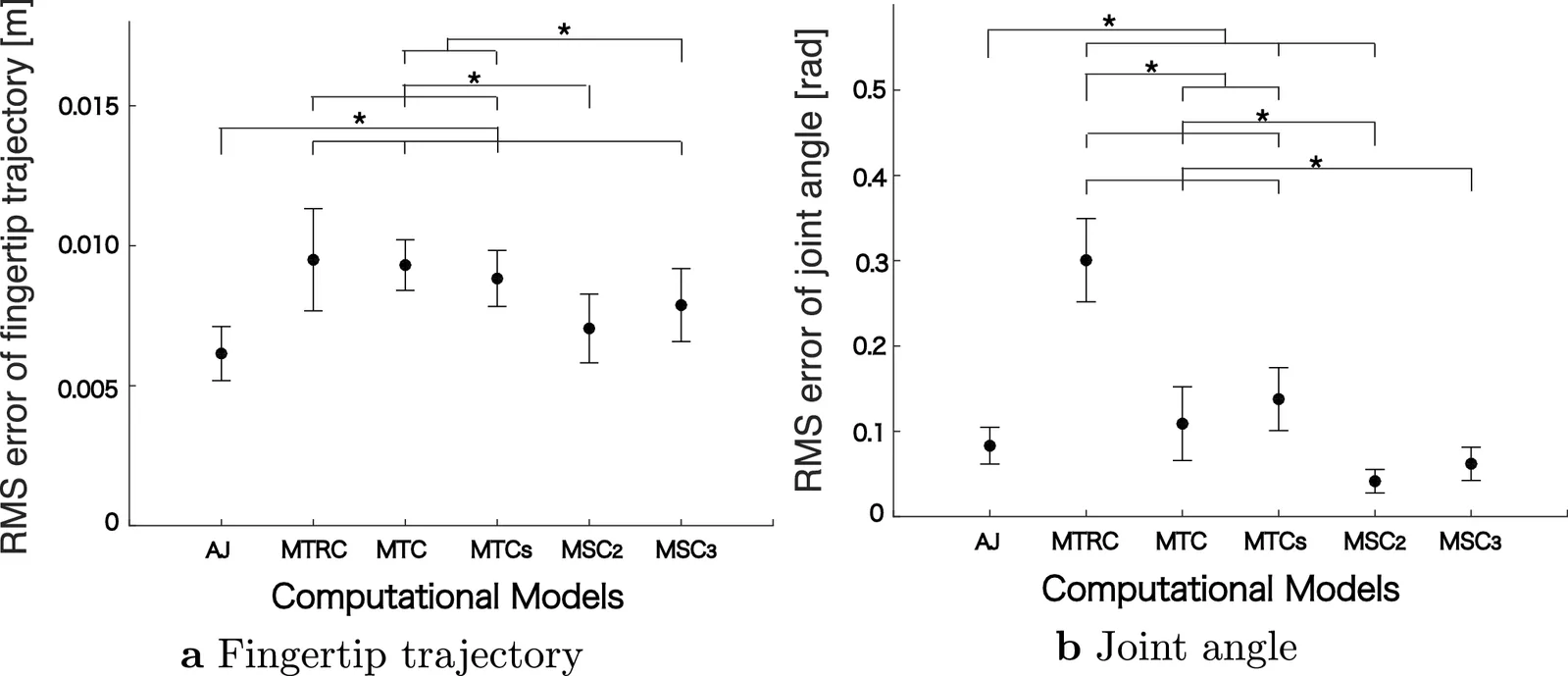
Model-dependent errors in trajectory and posture reproduction. Vertical bars are the standard deviation. Horizontal connectors indicate significant pairwise differences. (Muscle selection: S22. PCSA: PCSA1. *: p< 0.05)

**Fig. 9:**
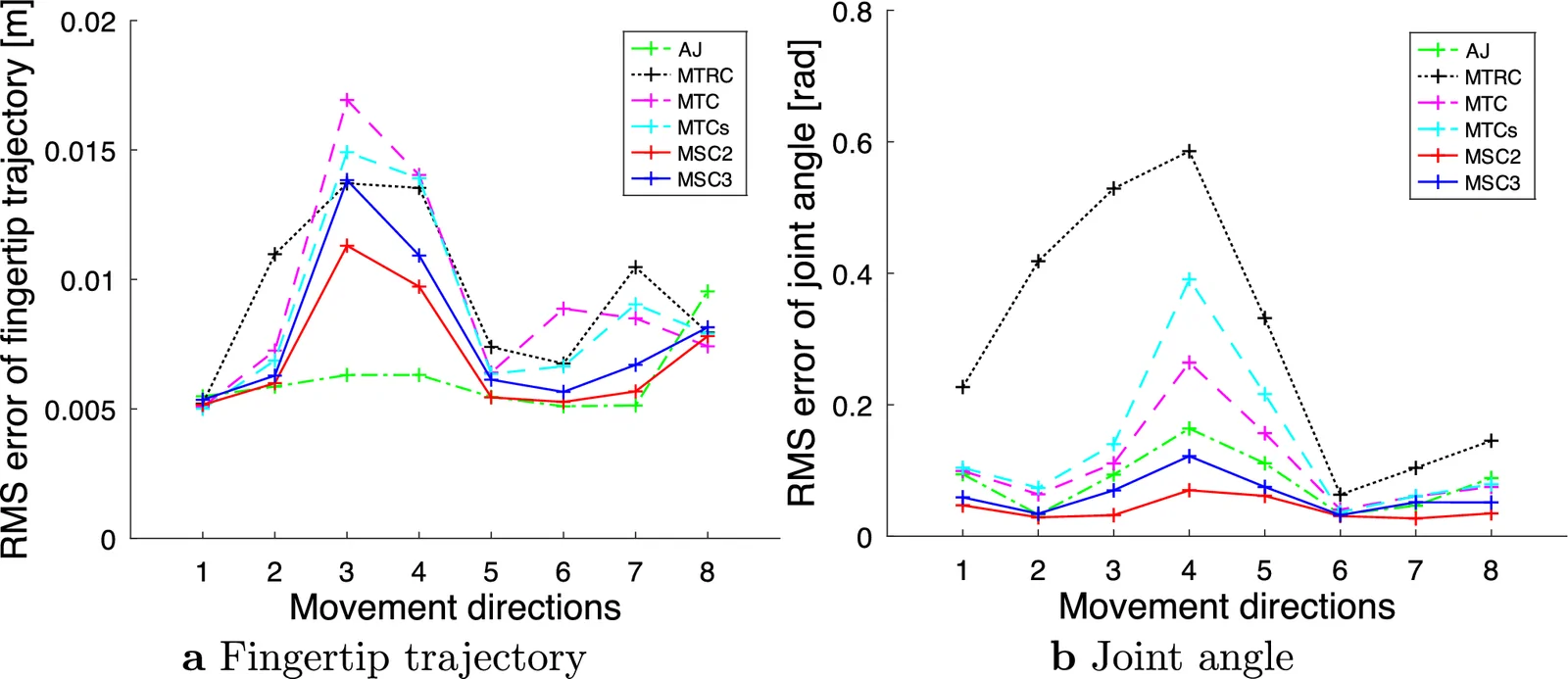
RMS errors for each movement direction. (Muscle selection: S22. PCSA: PCSA1)

**Fig. 10:**
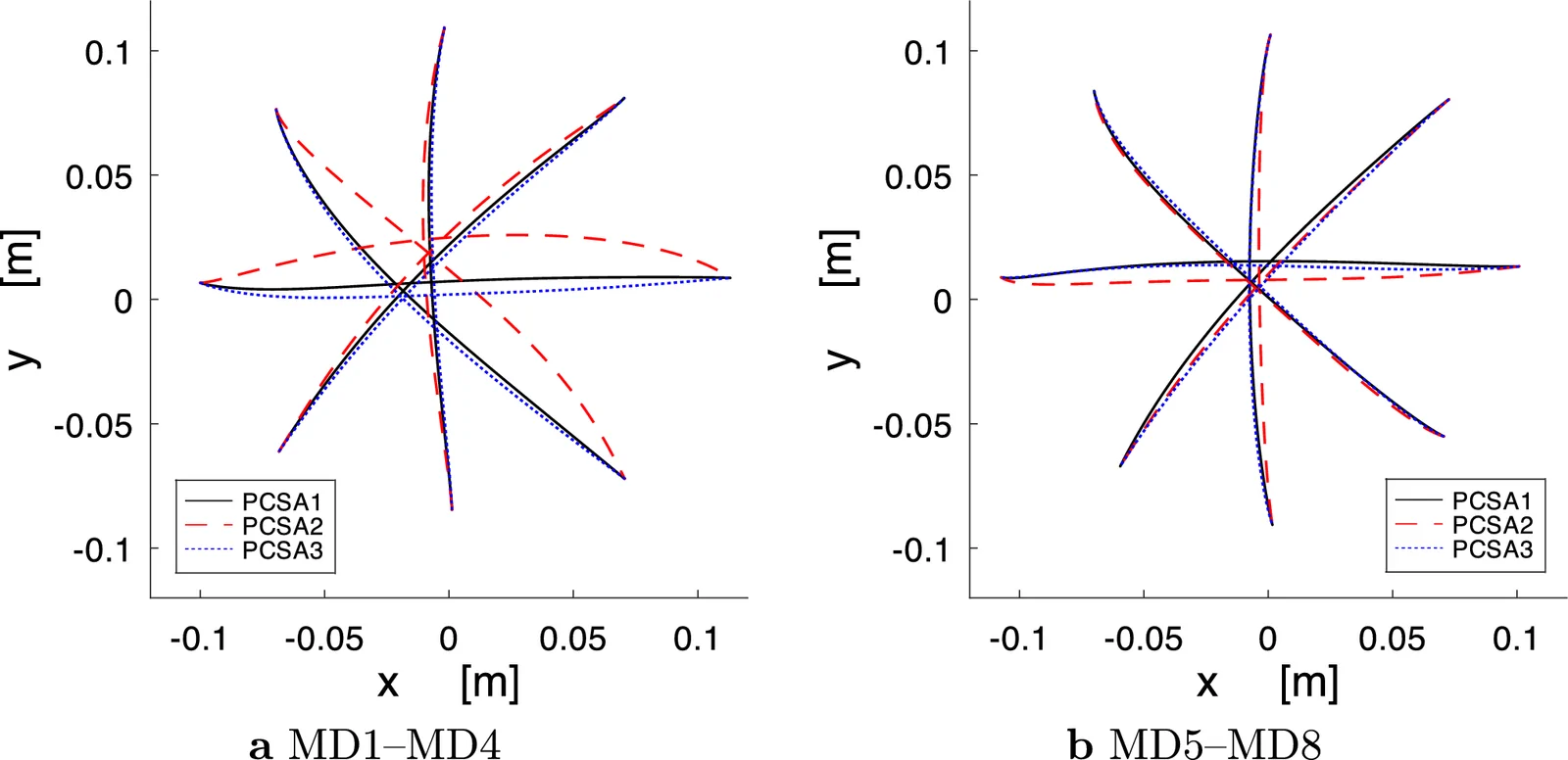
Influence of PCSA on the optimal fingertip paths. (Computational model: $\text {MSC}_2$, Muscle selection: S22)

**Fig. 11:**
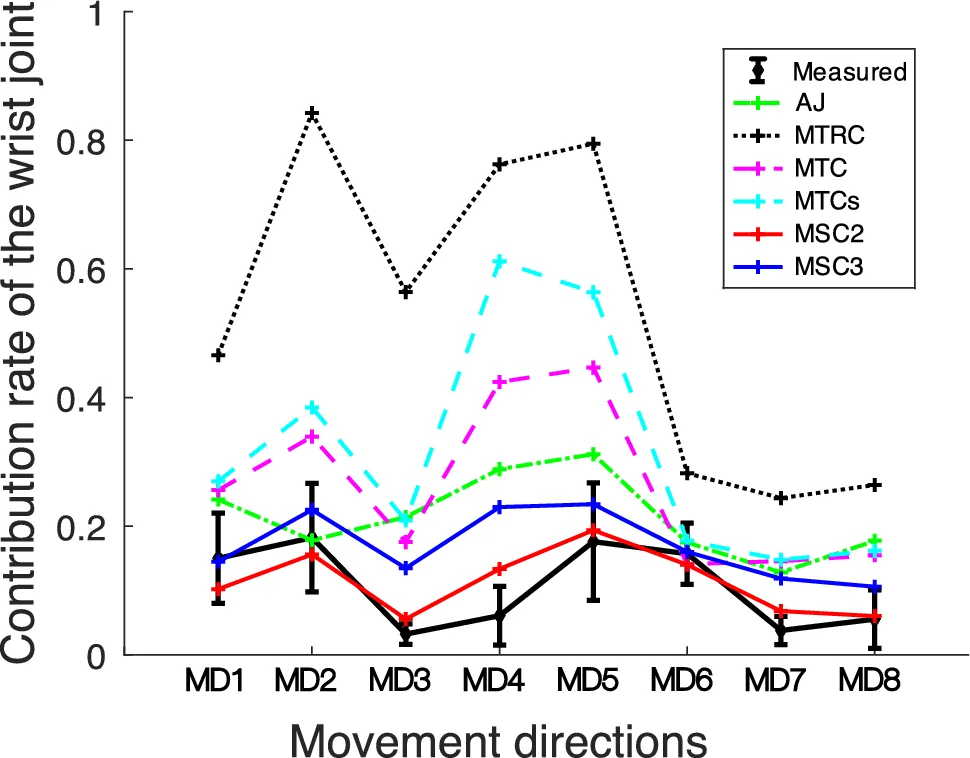
Contribution rate of the wrist joint. Vertical lines indicate the standard error of the mean and are shown only for the measured data; error bars for the computational models are omitted for clarity. (Muscle selection: S22, PCSA: PCSA1)

As shown in Fig. 8a, the RMS errors of the fingertip trajectory were smallest for the AJ and $\text {MSC}_2$ models, followed by the $\text {MSC}_3$ model. The direction-specific errors varied across models (Fig. 9a). $\text {MSC}_2$ produced larger errors in MD3, MD4, and MD8. The AJ model produced smaller errors in MD3 and MD4, but a larger error in MD8. In the remaining directions, both models showed similarly small errors. When the $\text {MSC}_2$ and $\text {MSC}_3$ models were compared, $\text {MSC}_3$ produced slightly larger errors in all directions, with the difference again being most pronounced in MD3 and MD4. As shown in Fig. 8b, the RMS errors of the arm posture were smallest for $\text {MSC}_2$, followed by the AJ and $\text {MSC}_3$ models. Direction-specific analysis (Fig. 9b) further showed that both the MTC and $\text {MTC}_s$ models produced large posture errors in MD4, with the error being larger for the $\text {MTC}_s$ model. In contrast, the $\text {MSC}_2$ and $\text {MSC}_3$ models showed small posture errors in all movement directions, and $\text {MSC}_2$ consistently produced smaller errors than $\text {MSC}_3$.

The effects of PCSA and muscle selection were also examined (see Supplementary Materials for the results). As shown in Fig. 10, the optimal fingertip trajectories generated by the $\text {MSC}_2$ model varied across the three PCSA sets. However, when the RMS error of the optimal fingertip trajectory was aggregated across all movement directions, no substantial differences were found among the three PCSA sets. The errors in the MTC model also showed little difference among the PCSA sets. For arm posture, the error obtained with PCSA2 in the $\text {MSC}_2$ model was slightly larger than those obtained with the other PCSA sets. For the MTC model, the error obtained with PCSA3 was smaller than those obtained with the other PCSA sets. For muscle selection, the $\text {MSC}_2$ and MTC models were also evaluated under the four muscle-selection conditions listed in Table 2. The fingertip-trajectory and arm-posture errors of $\text {MSC}_2$ changed only slightly across the muscle-selection conditions. For the MTC model, the fingertip-trajectory error was somewhat larger for muscle selection S12 and slightly smaller for S21 than for the other conditions. For arm posture, the errors were slightly larger for S11 and S21. Therefore, the arm movements predicted by the $\text {MSC}_2$ model were not substantially affected by the choice of PCSA dataset or by muscle selection with different numbers and types of muscles.

### Wrist-joint contribution for different movement directions

The measured arm postures included directions with substantial wrist rotation and directions with almost no wrist rotation (Fig. 5). As shown in Fig. 11, the contribution rate of the wrist joint, $C_w$, in the measured arm postures exhibited a bimodal profile, with peaks in MD2 and MD5. In directions where $C_w$ was small (MD3, MD4, MD7, and MD8), the shoulder-rotation angle was larger than that in the other movement directions. All computational models also produced a bimodal pattern of $C_w$, but the magnitude of $C_w$ and the directions of its peaks differed across models. The MTRC model overestimated $C_w$. The MTC and $\text {MTC}_s$ models reproduced the overall waveform shape reasonably well, but they overestimated $C_w$ in all directions except MD6, leading to excessive wrist rotation. The $\text {MTC}_s$ model produced larger errors than MTC, and its peak directions were slightly shifted. In the AJ model, the peak and trough directions were not well aligned with the measured data, and $C_w$ was large in all directions except MD2 and MD6. The $\text {MSC}_2$ model reproduced the bimodal pattern and the peaks in MD2 and MD5 more closely than the other models. The $\text {MSC}_3$ model showed a waveform shape similar to that of the measured data, but the errors in $C_w$ were larger than those of the $\text {MSC}_2$ model, particularly in MD3 and MD4.

### Muscle tensions selected by the computational models

**Fig. 12:**
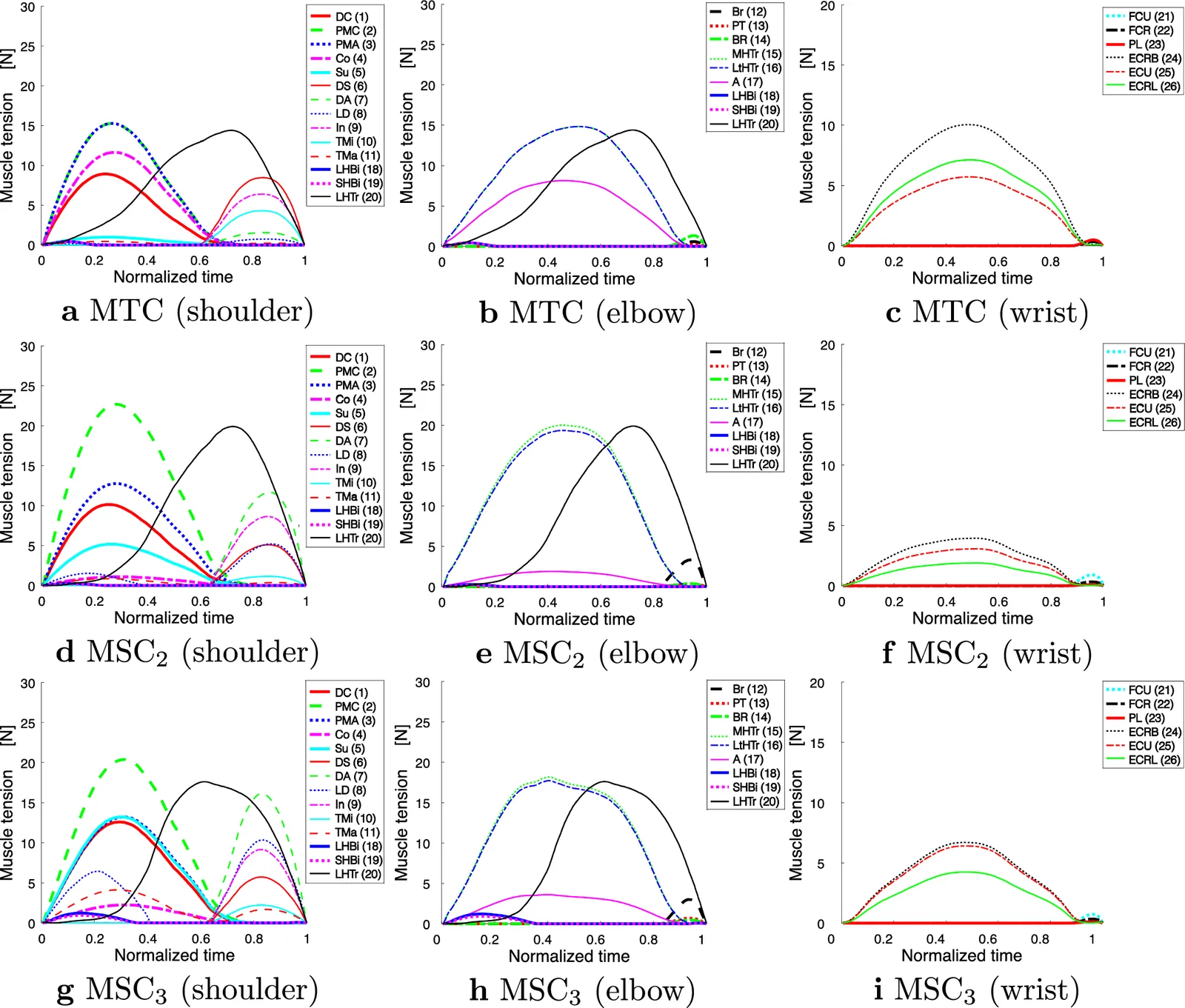
Muscle tensions predicted by the MTC, $\text {MSC}_2$, and $\text {MSC}_3$ models. Time was normalized to the movement time of each trial. Muscle tensions were averaged across all participants. (Muscle selection: S22, PCSA: PCSA1, Movement direction: MD8)

Examples of the muscle tensions predicted during movement by the MTC, $\text {MSC}_2$, and $\text {MSC}_3$ models are shown in Fig. 12. Because the temporal profiles of muscle tension were broadly similar across participants, the muscle tensions were averaged after normalizing time by the movement duration of each trial. To compare muscle recruitment across all movement directions, the activation rate of each muscle was computed under muscle selection S22 and PCSA1 (Table 4). Muscle recruitment in the MTC model differed from that in the other models for many muscles. The $\text {MSC}_3$ model recruited more muscles, including muscles that were rarely active in the $\text {MSC}_2$ model.

**Fig. 13:**
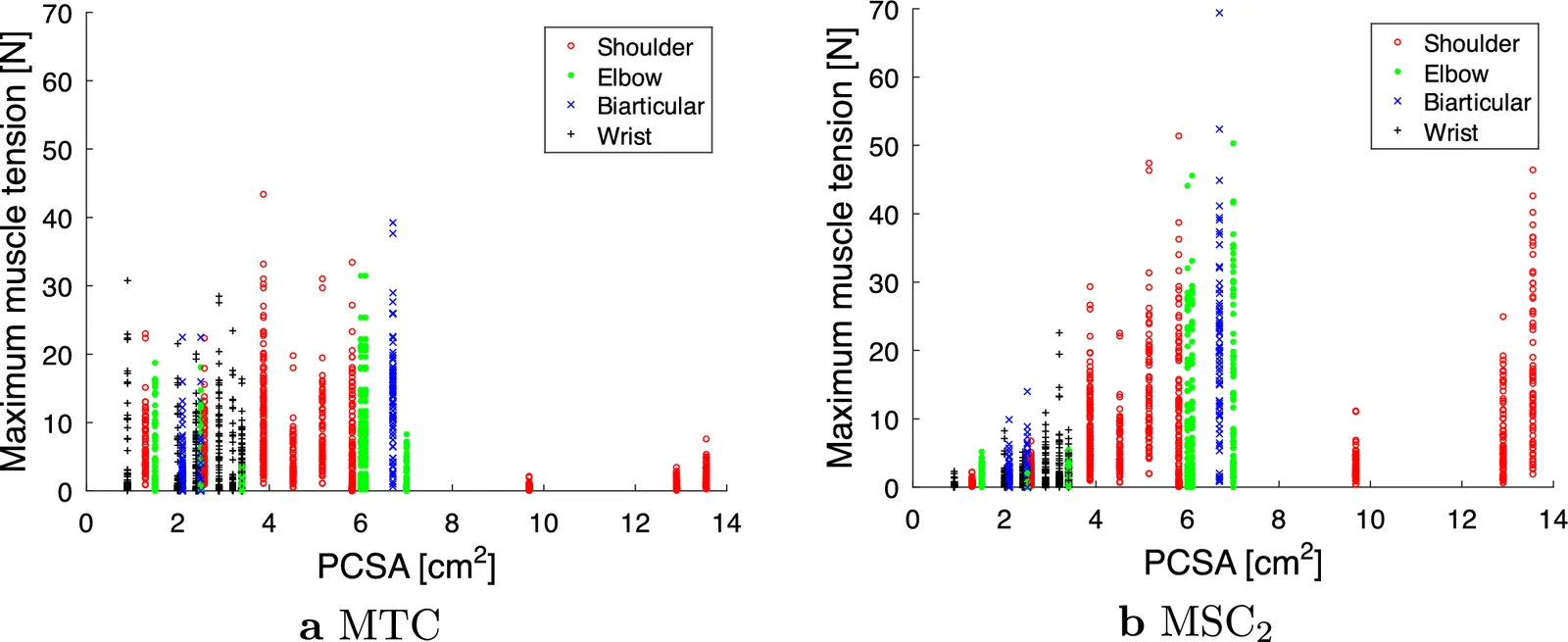
Relation between PCSA and peak predicted tension. Each point corresponds to one muscle in one movement trial. (Muscle selection: S22, PCSA: PCSA1)

**Fig. 14:**
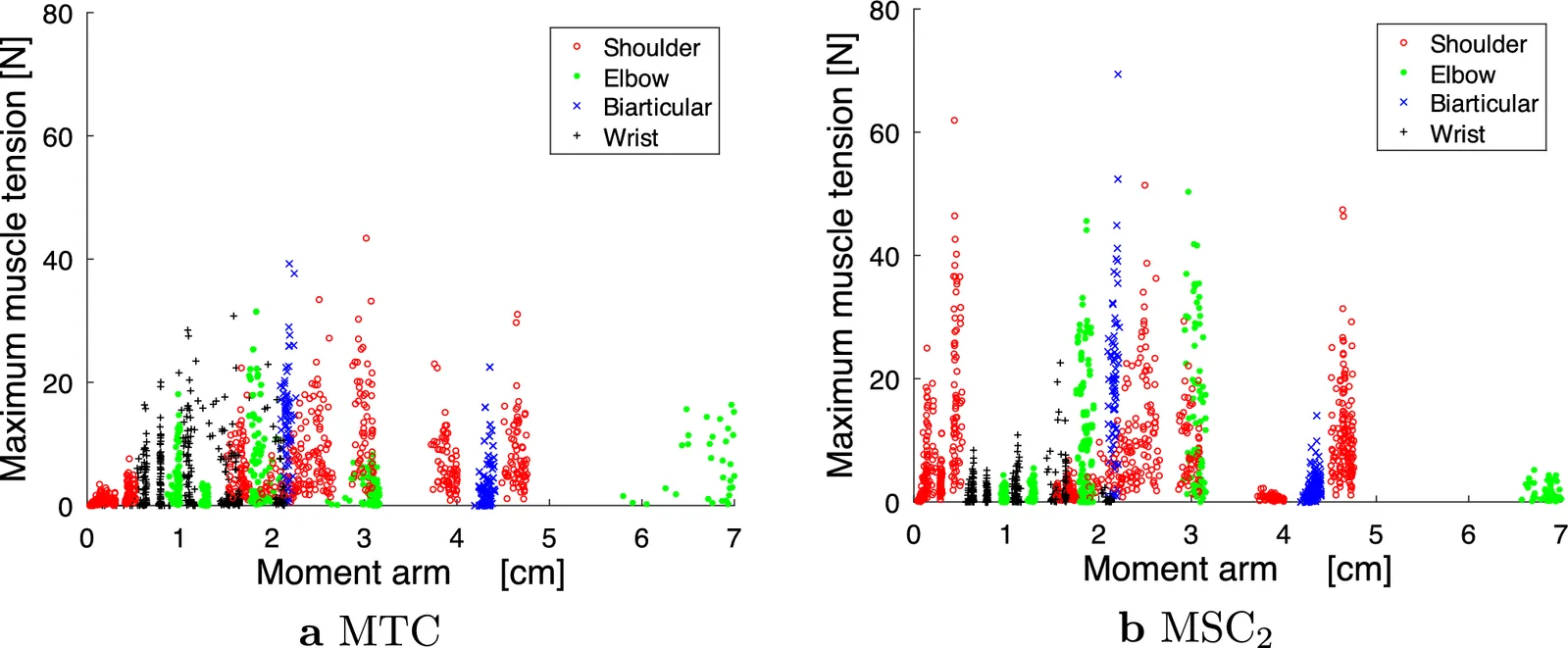
Relation between trial-averaged moment arm and peak predicted tension. The moment-arm magnitude was defined as the mean absolute moment arm during each movement trial, and peak tension was computed separately for each muscle. (Muscle selection: S22, PCSA: PCSA1)

To examine the strategy by which each model determined muscle tension, Figs. 13 and 14 show the relationships between peak muscle tension and PCSA and between peak muscle tension and moment arm, respectively. The moment-arm magnitude was defined as the mean absolute moment arm during each movement trial. The $\text {MSC}_2$ and MTC models suggested different recruitment principles. In the MTC model, no clear relationship was found between PCSA and the largest peak tensions. A notable feature of this model, however, was that muscles with very small PCSAs, such as muscles 4, 10, 14, 17, 18, 19, 22, 23, 24, and 26, tended to generate larger tensions in the MTC model than in the $\text {MSC}_2$ model, whereas the tensions of muscles with large PCSAs, such as muscles 5, 7, and 8, remained small. In the $\text {MSC}_2$ model, the largest peak tension decreased with decreasing PCSA for muscles with PCSAs below 7 $\text {cm}^2$, indicating that peak tension was related to PCSA. In addition, muscles with small PCSAs at each joint (e.g., muscles 4, 14, and 23) were rarely activated, whereas muscles with large PCSAs tended to generate larger tensions than in the MTC model. A different tendency was observed with respect to moment arm. In the MTC model, the largest peak tension decreased as moment arm decreased, particularly when the moment arm was below 3 cm. In the $\text {MSC}_2$ model, however, no clear relationship was found between peak muscle tension and moment arm.

## Discussion

### Comparison of computational model performance

Most previous studies of reaching movements have examined point-to-point reaches over distances of approximately 30–50 cm (e.g., Hogan 1984; Uno et al. 1989; Nakano et al. 1999; Katayama 2025). In contrast, the present study focused on shorter reaches (20 cm), so that all participants, including shorter individuals, could perform movements in eight directions within the reachable workspace. Although differences among computational models might be expected to diminish for shorter reaches, the present results showed that clear differences remained, particularly in the reproduction of arm posture.

Comparison of the computational models further showed that small errors in fingertip trajectory and arm posture do not necessarily guarantee accurate reproduction of the direction-dependent contribution of the wrist joint. The AJ model produced small errors in fingertip trajectory and arm posture, but its direction-dependent wrist contribution did not match the characteristic pattern observed in the measurements. By contrast, although the $\text {MSC}_2$ model showed relatively large fingertip-trajectory errors in MD3 and MD4, it yielded small posture errors across all movement directions and reproduced the measured wrist contribution more accurately than the other models. The $\text {MSC}_3$ model showed qualitative tendencies similar to those of the $\text {MSC}_2$ model, but the trajectory and posture errors were slightly larger, and the mismatch in wrist contribution was also greater. These results indicate that the apparent reproducibility of human arm movements depends on the evaluation metric used—fingertip trajectory, arm posture, or wrist contribution—and therefore should be assessed from multiple perspectives.

The optimal fingertip trajectories and arm postures predicted by each computational model depend on the joint-viscosity parameters. Katayama (2025) evaluated the $\text {MSC}_2$ and TC models under five joint-viscosity conditions and reported that $\text {MSC}_2$ was largely insensitive to joint viscosity, whereas the TC model showed larger errors in fingertip trajectories and arm postures under low-viscosity conditions but reproduced them more accurately under high-viscosity conditions. In the present study, additional analyses were conducted to examine the viscosity dependence of the $\text {MSC}_2$ and MTC models (see Supplementary Materials for the results). The influence of joint viscosity on $\text {MSC}_2$ was again small. For the MTC model, the mismatch with the measurements was reduced under higher-viscosity conditions, similar to the tendency observed for the TC model. Therefore, it is important to evaluate the models using joint-viscosity values that are as close as possible to the true values for the human arm. Although many previous studies have reported estimates of wrist-joint viscosity (e.g., Sinkjær and Hayashi 1989; Falzarano et al. 2021), the reported range is broad. This variability likely reflects differences in cocontraction of flexor and extensor muscles, the presence or absence of grasping, and the method used to apply perturbations, all of which can alter viscosity during movement. Reported wrist-joint viscosity values therefore range widely, from nearly zero to 0.6 $\mathrm {N\,m\,s/rad}$. In the present study, the baseline wrist-joint viscosity used in the expanded arm models was set near the middle of the reported range. In addition, to prevent participants from exerting excessive force during movement measurements, they completed sufficient practice beforehand, including several wrist-movement exercises, and performed a brief wrist movement immediately before each trial (corresponding to a so-called waggle). These procedures likely reduced cocontraction, suggesting that joint viscosity during movement was not excessively high.

Changing the joint-viscosity value also changes the muscle-tension profiles of agonist and antagonist muscles. In general, higher viscosity increases viscous force; compensating for this requires greater agonist tension and reduced antagonist tension. As shown in Fig. 12, antagonist tension at the elbow and wrist was already small under the viscosity used in the present study. Because this tendency was observed across all movement directions, the joint viscosity adopted here may even have been somewhat overestimated. If viscosity were increased further, antagonist tension would likely decrease even more, approaching a pattern dominated by agonist tension alone; however, such a pattern would not be consistent with the agonist–antagonist activation typically observed in human movement. From the standpoint of joint viscosity, the values used in the present study—or possibly smaller values—therefore appear plausible. This consideration also suggests that the TC and MTC models should be interpreted cautiously, because their ability to reproduce the measured three-joint point-to-point reaching movements may depend strongly on the assumed viscosity values.

**Table 4:** Percentage (%) of muscles activated during optimal arm movements across all movement directions for each computational model. A muscle was considered active in a given trial if its tension was ≥5.0 N for shoulder muscles, ≥3.5 N for elbow and biarticular muscles, and ≥2.0 N for wrist muscles. (mean ± standard error of the mean, Muscle selection: S22, PCSA: PCSA1)

Muscle #	1	2	3	4	5	6	7	8
MTRC	29 ± 8	68 ± 13	50 ± 6	0 ± 0	6 ± 12	47 ± 14	85 ± 10	44 ± 13
MTC	33 ± 17	69 ± 16	69 ± 16	56 ± 25	0 ± 0	79 ± 14	4 ± 8	0 ± 0
$\text {MTC}_s$	43 ± 22	88 ± 0	72 ± 14	0 ± 0	17 ± 19	54 ± 17	85 ± 13	47 ± 19
$\text {MSC}_2$	46 ± 24	92 ± 8	65 ± 21	0 ± 0	12 ± 20	68 ± 16	88 ± 10	56 ± 23
$\text {MSC}_3$	65 ± 21	88 ± 16	68 ± 21	0 ± 0	67 ± 20	69 ± 15	93 ± 6	86 ± 7

9	10	11	12	13	14	15	16	17
78 ± 13	0 ± 0	0 ± 0	65 ± 18	3 ± 8	3 ± 8	56 ± 16	54 ± 14	0 ± 0
68 ± 19	50 ± 16	0 ± 0	28 ± 10	1 ± 4	49 ± 7	61 ± 18	61 ± 18	46 ± 8
79 ± 14	0 ± 0	0 ± 0	71 ± 18	1 ± 4	1 ± 4	57 ± 12	57 ± 12	0 ± 0
82 ± 16	3 ± 8	0 ± 0	75 ± 21	11 ± 14	11 ± 14	58 ± 8	58 ± 8	1 ± 4
85 ± 13	24 ± 26	14 ± 16	72 ± 21	43 ± 9	22 ± 11	65 ± 14	65 ± 14	22 ± 10

18	19	20	21	22	23	24	25	26
25 ± 17	10 ± 16	88 ± 6	50 ± 14	40 ± 8	29 ± 6	71 ± 8	74 ± 9	65 ± 10
33 ± 18	33 ± 18	90 ± 5	29 ± 13	28 ± 10	33 ± 20	61 ± 9	56 ± 6	58 ± 8
31 ± 20	12 ± 16	90 ± 5	47 ± 19	33 ± 8	28 ± 5	56 ± 9	54 ± 6	54 ± 6
28 ± 19	12 ± 20	90 ± 5	36 ± 19	17 ± 12	3 ± 8	46 ± 14	32 ± 10	18 ± 18
49 ± 21	32 ± 20	93 ± 6	43 ± 17	32 ± 9	11 ± 16	57 ± 6	57 ± 6	56 ± 6

### Limitations of the first-stage optimization

Under the constraints imposed in the first-stage optimization, the arm is stationary at the start and end points. These boundary conditions require the joint torques at both endpoints to be zero; consequently, the first-stage optimization yields zero muscle tensions at the beginning and end of the movement (Fig. 12). Under these constraints, the predicted cocontraction of flexor and extensor muscles during movement tends to be limited. In the present experiment, participants practiced extensively so that they could perform the reaching movements smoothly while reducing excessive cocontraction. In addition, the movement speed was set slightly lower than that of typical everyday arm movements, a relatively large target area was used, and participants were instructed not to force the fingertip into the target at the end of the movement. Because fingertip overshoot was very small in the measured movements, these endpoint constraints appear to be reasonable approximations for the present task.

In human reaching movements, however, triphasic muscle activity is often observed: an initial agonist burst is followed by antagonist activity and then by a second agonist burst (e.g., Kambara et al. 2013). Because the optimization used in the present study imposes zero muscle tension at the endpoint, the muscle-tension profiles predicted by the MSC and MTC models do not exhibit such triphasic patterns. Nevertheless, the present feedforward-planning framework could in principle be extended to account for triphasic activity. Two possible approaches can be considered. The first is to combine the present feedforward-planning model with feedback control driven by movement errors, under the assumption that a mismatch between the internal dynamics model used for planning and the actual arm dynamics causes the realized trajectory to deviate from the optimal trajectory. The second is to introduce signal-dependent noise in muscle tension, as in Harris and Wolpert (1998), and to combine it with feedback control that reduces the resulting trajectory deviations.

### Muscle recruitment

This section discusses the strategy by which each computational model determines muscle recruitment, represented here by the muscle-tension distribution. This evaluation is central to the present study and distinguishes it from Katayama (2025), because the present 19–26-muscle model with joint-angle-dependent moment arms allows anatomical muscle recruitment to be examined, rather than only trajectory and posture reproduction.

Compared with the MTC model, the $\text {MSC}_2$ and $\text {MSC}_3$ models tended to suppress activation of muscles with small PCSAs and to recruit muscles with larger PCSAs more strongly. This tendency is consistent with the fact that, in both models, the objective function directly affects muscle-tension distribution through muscle stress (muscle tension divided by PCSA), thereby favoring solutions that avoid imposing high stress on muscles with small PCSAs. From a mechanical standpoint, however, a muscle with a smaller moment arm produces less torque for the same muscle tension, so selecting muscles with larger moment arms is more mechanically efficient. Indeed, the $\text {MSC}_2$ model also tended to avoid recruiting muscles with small moment arms, but muscles 5, 7, and 8 were recruited despite their relatively small moment arms because they have large PCSAs in PCSA1. This result suggests that, in the MSC framework, PCSA strongly influences muscle recruitment in addition to moment arm.

When muscle recruitment under PCSA1 and PCSA3 was compared for the $\text {MSC}_2$ model, muscles with large PCSAs in PCSA3, such as muscle 1, tended to be recruited more frequently, whereas muscles with small PCSAs, such as muscles 2, 6, 7, and 8, were recruited less frequently. Thus, in the $\text {MSC}_2$ model, the set of recruited muscles and their recruitment levels varied depending on the PCSA values used in the optimization. Furthermore, S11, S12, and S21 in Table 2 include fewer muscles than S22; several muscles included in S22 are omitted or included only as supplementary muscles in these muscle selections. These muscles have short moment arms, small PCSAs, and/or possible roles in joint stabilization. However, as shown in Table 4, many of these muscles were in fact recruited. This suggests that muscles with short moment arms or small PCSAs should be considered rather than excluded a priori. Therefore, within the assumptions of the model, the muscle-recruitment strategy of the MSC framework is physiologically interpretable because it takes both PCSA and moment arm into account and may reduce excessive loading of muscles with smaller PCSAs. However, whether the muscle-recruitment patterns predicted by the MSC framework correspond to actual human muscle recruitment during reaching has not been directly evaluated. Direct evaluation would require comparing the predicted muscle-recruitment patterns with EMG recorded during the same reaching task, together with participant-specific estimates of PCSA and moment arms.

Compared with the $\text {MSC}_2$ model, the $\text {MSC}_3$ model still tended not to recruit muscles with extremely small PCSAs, but it recruited more muscles, including some that were rarely recruited by $\text {MSC}_2$. This tendency is consistent with the fact that the $\text {MSC}_3$ model uses an objective function that includes the cube of muscle stress (p=3), so the penalty for concentrating high stress in a specific muscle is larger than in $\text {MSC}_2$ (p=2), making it easier to distribute muscle tension across multiple muscles (Crowninshield and Brand 1981). In fact, the number of muscles with activation rates above 10 % was 21 for both the MTC and $\text {MSC}_2$ models, whereas it increased to 25 for the $\text {MSC}_3$ model, meaning that nearly all muscles except muscle 4 under muscle-selection condition S22 were recruited. Therefore, the tendency of muscle recruitment varies depending on the form of the objective function in the MSC models (p=2 or p=3) and on the PCSA and moment-arm values used in the optimization.

Overall, movement-level predictions of the $\text {MSC}_2$ model were relatively robust, whereas its muscle-recruitment predictions were more sensitive to PCSA and moment-arm values. This difference indicates that reproduction of fingertip trajectories and arm postures alone is insufficient for evaluating muscle-level computational models. The validity of such models should ideally be assessed not only in terms of errors in fingertip trajectory, arm posture, and posture-related characteristics such as the wrist-joint contribution rate, $C_w$, introduced in this study, but also in terms of muscle recruitment, for example using electromyographic measurements during movement. However, such evaluation is challenging because predicted recruitment patterns may vary depending on the PCSA and moment-arm values assigned to individual muscles in each participant.

In addition, the present models do not explicitly account for task-dependent agonist–antagonist cocontraction. Such cocontraction has been reported when stability against external perturbations is required (Burdet et al. 2001), in responses to joint perturbations (Lewis et al. 2010; Armstrong et al. 2025), under increased accuracy demands (Gribble et al. 2003), and during motor learning (Osu et al. 2002). Because cocontraction can alter muscle recruitment, the recruitment patterns predicted by the present models may differ from actual human muscle recruitment under these conditions. Accordingly, the present predictions should be interpreted primarily in relation to the unperturbed reaching task examined here, for which the experimental procedures were designed to reduce excessive cocontraction, as described in Sect. 5.2.

### Relation to neuromuscular synergy theories

The present results are relevant to neuromuscular synergy theories. Muscle synergies are often extracted from electromyographic (EMG) signals using non-negative matrix factorization, and previous studies have suggested that complex upper-limb movements can be represented by combinations of a relatively small number of coordinated muscle-activation patterns (e.g., d’Avella et al. 2006; Bizzi and Cheung 2013). However, synergy extraction does not necessarily capture all task-relevant muscle activity, because residual EMG components may still influence task performance (Barradas et al. 2020).

The comparison between the $\text {MSC}_2$ and $\text {MSC}_3$ models provides a useful computational perspective on this issue. The $\text {MSC}_2$ model reproduced fingertip trajectories, arm postures, and the wrist-joint contribution rate more accurately than $\text {MSC}_3$ and, as shown in Table 4, recruited fewer muscles. Conversely, under muscle-selection condition S22, the $\text {MSC}_3$ model recruited almost all muscles, but its reproduction accuracy for these components of arm movement was slightly lower than that of the $\text {MSC}_2$ model. These results suggest that human reaching movements do not simply reflect maximally distributed muscle activity. Rather, coordinated muscle activity resembling muscle synergies may emerge from an optimization process that constrains muscle stress to change smoothly while taking into account muscle-specific differences in PCSA and moment arms. Thus, the $\text {MSC}_2$ model may provide a candidate computational mechanism for generating coordinated muscle-recruitment patterns without assuming predefined fixed muscle synergies.

Because EMG signals were not recorded in the present study, however, muscle synergies could not be extracted from actual muscle activity. Therefore, an additional muscle-synergy analysis was not performed. Even if synergies were extracted from the muscle tensions determined by each computational model, it would be difficult to draw direct conclusions about physiological muscle synergies. Future studies should record EMG signals during reaching movements under the same movement conditions as those used in the present study and examine whether the recruitment patterns predicted by the $\text {MSC}_2$ model correspond to extracted muscle synergies and how such patterns depend on PCSA and moment-arm properties.

### Feedforward planning and optimal feedback control

The present model is also relevant to the distinction between feedforward planning and optimal feedback control (OFC). In feedforward planning, smoothness-based computational models, such as the MTC and MSC models, define optimal movement trajectories and can therefore predict internal variables such as joint torque, muscle tension, and muscle recruitment. However, because these models do not include an explicit feedback mechanism and movement duration must be specified as a boundary condition, they cannot fully account for online correction of movement errors or trial-to-trial variability.

By contrast, OFC emphasizes flexible online regulation based on sensory feedback and the minimum intervention principle, whereby deviations in task-irrelevant dimensions are tolerated, whereas deviations related to the behavioral goal are corrected (e.g., Todorov and Jordan 2002; Diedrichsen et al. 2010; Kambara et al. 2021). Although OFC has these advantages, standard feedback-dependent OFC frameworks may have difficulty accounting for goal-directed movements after deafferentation in monkeys, because such movements can be generated even when sensory feedback from the limb is severely impaired (Polit and Bizzi 1979). In addition, how high-dimensional muscle redundancy is resolved in a three-joint arm model with muscle-specific PCSAs, joint-angle-dependent moment arms, and up to 26 muscles remains an open question.

Thus, feedforward planning and OFC should be regarded not as mutually exclusive alternatives but as complementary frameworks. The MSC framework can determine muscle-tension distributions in a feedforward manner by taking into account physiological properties of the arm musculoskeletal system, such as PCSA and joint-angle-dependent moment arms. Because these distributions avoid excessive loading of muscles with smaller PCSAs and tend to assign larger tensions to muscles with larger PCSAs, they are physiologically interpretable. During the execution of movements generated by feedforward planning, as discussed in Sect. 5.2, feedback control may then correct movement errors online. In this sense, the MSC framework may complement OFC by specifying how anatomically constrained muscle redundancy is resolved in the feedforward component of arm movement generation.

### Limitations and future directions

Several limitations of this study should be noted. First, all participants were right-handed healthy young adults; therefore, the generalizability of the present findings to other populations, such as left-handed individuals or older adults, remains unclear. Second, the experimental task was limited to reaching movements in the horizontal plane. Third, the solution obtained with the two-stage optimization framework is not guaranteed to coincide with the solution that would be obtained by directly minimizing the second-stage objective function alone, such as $C_{MSC_2}$. Fourth, the validity of the muscle recruitment patterns predicted by the MSC framework was not directly verified by EMG. Future studies should combine the present eight-direction reaching task with EMG measurements, estimate participant-specific anatomical parameters where possible, and examine whether muscle synergies or other EMG-derived coordination patterns correspond to the recruitment patterns predicted by the MSC framework. Overall, the present findings support the $\text {MSC}_2$ model as a muscle-level optimization model that links task-space behavior, joint-space coordination, and physiologically interpretable muscle recruitment.

## Supplementary Information

Below is the link to the electronic supplementary material.

Supplementary file 1 (pdf 224 KB)

Supplementary file 2 (pdf 26 KB)

Supplementary file 3 (pdf 84 KB)

## Data Availability

The datasets generated herein are available from the corresponding author upon reasonable request.

## Appendix A Polynomial coefficients for calculating muscle moment arms

**Table 5:** Polynomial coefficients for calculating muscle moment arms as functions of joint angle. a(θ) is the moment arm (cm), and θ is the joint angle (degrees). S, E, and W denote the shoulder, elbow, and wrist joints, respectively

#		$a_0$	$a_1$	$a_2$	$a_3$	$a_4$	$a_5$	$a_6$	$a_7$
1	S	5.031	−8.503e−2	7.429e−4	−2.825e−6				
2	S	3.329	7.088e−2	−9.996e−4	3.061e−6				
3	S	3.329	7.088e−2	−9.996e−4	3.061e−6				
4	S	2.479	1.975e−2	6.727e−4	−1.191e−5	3.836e−8			
5	S	5.084e−1	−8.177e−3	7.330e−5					
6	S	−5.385e−1	−1.074e−1	1.307e−3	−3.650e−6				
7	S	−6.235e−1	2.006e−2	−4.431e−4	2.428e−6				
8	S	3.808e−1	−9.496e−3						
9	S	−7.229e−1	−1.066e−1	1.690e−3	−6.530e−6				
10	S	−8.513e−1	−5.422e−2	9.190e−4	−3.824e−6				
11	S	2.987e−2	8.751e−3	−1.530e−4					
12	E	5.549e−1	2.308e−2	2.343e−4	−2.053e−6				
13	E	7.187e−1	9.990e−3	−1.556e−3	7.163e−5	−1.322e−6	1.202e−8	−5.419e−11	9.680e−14
14	E	1.949	1.668e−2	1.008e−3	−6.517e−6				
15	E	−2.329	−3.028e−2	1.289e−3	−1.909e−5	1.328e−7	−3.517e−10		
16	E	−2.329	−3.028e−2	1.289e−3	−1.909e−5	1.328e−7	−3.517e−10		
17	E	−5.345e−1	−2.284e−2	8.430e−4	−1.433e−5	1.045e−7	−2.731e−10		
18	S	2.479	1.975e−2	6.727e−4	−1.191e−5	3.836e−8			
18	E	1.466	4.532e−2	1.805e−4	−2.988e−6				
19	S	2.479	1.975e−2	6.727e−4	−1.191e−5	3.836e−8			
19	E	1.466	4.532e−2	1.805e−4	−2.988e−6				
20	S	−2.931	7.776e−3	1.031e−5					
20	E	−2.329	−3.028e−2	1.289e−3	−1.909e−5	1.328e−7	−3.517e−10		
21	W	1.625	2.688e−3	−8.302e−5					
22	W	1.508	3.615e−3	−1.011e−4					
23	W	2.100	3.615e−3	−1.011e−4					
24	W	−1.078	−2.532e−3	1.866e−5					
25	W	−6.083e−1	−3.649e−3	9.293e−5					
26	W	−7.961e−1	−8.920e−5	3.269e−5	−4.773e−7				

The moment arm, a(θ), was modeled using an *n*th-order polynomial: $a(\theta )=a_0+a_1\theta +a_2\theta ^2+a_3\theta ^3+\cdots +a_n\theta ^n$, where a(θ) is the moment arm (cm) and θ is the joint angle (degrees). The coefficients for the shoulder joint were derived from Kuechle et al. (1997), and those for the elbow and wrist joints were derived from Pigeon et al. (1996). Coefficients for biarticular muscles spanning the elbow joint were also derived from Pigeon et al. (1996). For the pronator teres (PT), the measurement results from Amis et al. (1979) were used. Joint angles of $\text {0}^\circ$ at the shoulder, elbow, and wrist indicate that the upper arm points to the right, the upper arm and forearm are aligned (elbow fully extended), and the forearm and hand are aligned, respectively. Polynomial degrees were chosen to be as low as possible while still reproducing the measurement data, and the coefficients were estimated by least-squares fitting.

## Code Availability

The code generated in this study is available from the corresponding author upon reasonable request.

## Materials Availability

Supplementary materials provide the dynamics of the three-joint arm, a description of the developed real-coded genetic algorithm (RCGA), and the results of analyses using three PCSA sets, four muscle-selection conditions, and different viscosity values.
