# Supplementary material for: Muscle-level evaluation of the minimum muscle-stress-change model in human three-joint reaching using anatomically expanded arm models
Source: Biol Cybern. 2026 Aug 22;120(5-6):29. doi: 10.1007/s00422-026-01056-2 (PMC13499790; doi:10.1007/s00422-026-01056-2)
Supplement: Supplementary file 1 [file 422_2026_1056_MOESM1_ESM.pdf]

Optimal movement selection by a real-coded genetic algorithm (RCGA)

Katayama, Masazumi

Division of Human and Artificial Intelligent Systems,

Graduate School of Engineering, University of Fukui, Japan

1 Introduction

In well-known genetic algorithms, the individuals are expressed as binary numbers. However, for optimal movement selection, it is necessary to consider constraints such as the achievability of hand positions and arm postures and the smoothness of an arm movement. In such a case, it is more advantageous to use a genetic algorithm with individuals that use real numbers.

In this study, we developed a real-coded genetic algorithm (RCGA) that selects the optimal hand trajectories and arm postures of three-jointed arm movements.

2 Method

■Flow of the algorithm

1. Set the initial values of the parameters such as the number of individuals of parents and children and so on)
2. Generate the initial parents
3. Generate four types of children from the parents
   - Crossover, Intermediate, Elite mutation, Mutation
4. Evaluate each individual of the children
5. Select two types of parents for the next generation from the children
   - Elite selection, Roulette selection

6. Stop if one of the termination conditions is satisfied, otherwise go to Step 3.

2.1 Set each initial value of the parameters

First, the number of individuals of the parents, children, elite mutation, mutation, elite selection and roulette selection, are determined. In addition, the maximum generation and each value of the following parameters are decided.

2.2 Expression of each individual

A time series from the initial state to the final state of an arm movement is equally divided into n-1 sections, and the i-th point is expressed such as x_i (see Harris and Wolpert, 1998).

The j-th individual that expresses a hand trajectory and arm postures on the horizontal plane is as follows:

$$\begin{aligned} \boldsymbol{x}^j &= \left(x_1^j, x_2^j, x_3^j, \ldots.., x_i^j, \ldots.., x_n^j\right), \\ \boldsymbol{y}^j &= \left(y_1^j, y_2^j, y_3^j, \ldots.., y_i^j, \ldots.., y_n^j\right), \\ \boldsymbol{\theta}^j &= \left(\theta_1^j, \theta_2^j, \theta_3^j, \ldots.., \theta_i^j, \ldots.., \theta_n^j\right). \end{aligned}$$

Here, $\boldsymbol{x}^j$ and $\boldsymbol{y}^j$ in the j-th individual are the coordinates of the x- and y-axes, respectively. $\boldsymbol{\theta}^j$ is joint angles of the wrist joint. In this algorithm, we use only the wrist angles as an individual because the joint angles of the shoulder and elbow joints can be uniquely calculated after the wrist angles are determined.

2.3 Generation of the initial parents

First, x_1^j, y_1^j, and θ_1^j of the initial state of an arm movement are set to their respective values of the measured arm movement, and x_n^j and y_n^j of the final state are also set to their respective values. In the other elements, the initial values of x_i^j, y_i^j, and θ_i^j are determined from normally distributed random values, and $\boldsymbol{x}^j$, $\boldsymbol{y}^j$ and $\boldsymbol{\theta}^j$ are smoothed using the smoothing method. Furthermore, these initial values are limited to the range of feasible arm movements.

x_1^j, y_1^j, and θ_1^j of the initial state of an arm movement are fixed to their respective

initial values, x_n^j and y_n^j of the final state are also fixed to their respective initial values, and θ_n^j of the final state is freely changed.

2.4 Generation of four types of children from the parents

The following explanation shows an example of $\boldsymbol{x}^j$.

■Crossover The m-th and n-th parents are selected.

$$\boldsymbol{x}^m = (x_1^m, x_2^m, x_3^m, ..., x_i^m, ..., x_n^m),$$
$$\boldsymbol{x}^n = (x_1^n, x_2^n, x_3^n, ..., x_i^n, ..., x_n^n).$$

The j-th child can be generated by interchanging the first and second halves of each parent.

$$\boldsymbol{x}^j = \left(x_1^m, x_2^m, ..., x_{k-1}^m, x_k^m, x_{k+1}^n, ...x_{n-1}^n, x_n^n\right).$$

Here, k is an integer part of $\frac{n}{2}$. The central elements of the child are smoothed out as follows:

$$\boldsymbol{x}_{k-1}^j = 0.75 * x_{k-1}^m + 0.25 * x_{k-1}^n,$$
$$\boldsymbol{x}_k^j = 0.5 * x_k^m + 0.5 * x_k^n,$$
$$\boldsymbol{x}_{k+1}^j = 0.25 * x_{k+1}^m + 0.75 * x_{k+1}^n.$$

■Intermediate The m-th and n-th parents are selected as described in the previous section. The j-th child can be generated as follows:

$$\boldsymbol{x}_i^j = \alpha_i \left(x_i^n - x_i^m\right) + x_i^m.$$

Here, $0 \leq \alpha_i \leq 1$. In this study, $\alpha_i = 0.5$.

■Elite mutation Some elite parents with lower evaluation values are selected. A few points of the parent are selected probabilistically, and random numbers are added to these points to generate the children. In the case that the i-th point of the k-th parent is selected probabilistically, a random number, ϵ_i, is added to the i-th point.

$$\boldsymbol{x}^k = \left(x_1^k, x_2^k, x_3^k, ..., x_i^k + \beta\epsilon_i, ..., x_n^k\right)$$

The points before and after the added random number are discontinuous, so they are smoothed.

β is a coefficient that determines the magnitude of the perturbation, and the value is changed as follows:

$$\beta = \alpha \exp(-\frac{g}{T}) + \beta_{min}$$

Here g is the generation and β_{min} is the minimum value of β. T and α are coefficients.

Note that the elements of x_1^j, y_1^j, θ_1^j, x_n^j and y_n^j are excluded, although θ_n^j changes by a random number.

■Mutation Some parents are selected probabilistically. Random numbers are added to the selected points of each parent, as in the elite mutation above.

2.5 Evaluation of each individual of the children

Each evaluation value of the children is calculated. The smaller the evaluation value, the better the child individual.

STEP1: Convert discrete elements of a child into time series data using a 5th spline

STEP2: Calculate the wrist joint position from the converted hand position and wrist angle

STEP3: Calculate the joint angles of the shoulder and elbow joints that achieve these positions (inverse kinematics)

STEP4: Calculate the joint torque at each time using the dynamics equation of a three-jointed arm (inverse dynamics)

STEP5: Determine the muscle tension at each time from the joint torque (first stage optimization)

STEP6: Calculate muscle stresses from the muscle tensions

STEP7: Calculate the evaluation value of each computational model (second stage optimization)

Minimum angular jerk model: Steps 1-3 and Step 7

Minimum torque change model: Steps 1-4 and Step 7

Minimum muscle stress change model: Steps 1-7

2.6 Selection of two types of parents for the next generation

Two types of parents for the next generation are selected from the children.

■Elite selection Some elite children with lower evaluation values are selected as the parents of the next generation.

■Roulette selection Some children are probabilistically selected as the parents of the next generation, and the probability, P_l, of the selection is determined by the evaluation value of each individual. I_l is the inverse of the evaluation value, C_l, of the l-th child individual.

$$P_l = \frac{I_l}{\sum_{l=1}^{N} I_l}$$

2.7 Termination conditions

The algorithm stops when one of the termination conditions is satisfied.

1. Maximum generation
2. The smallest evaluation value is less than the threshold.

3 Check the algorithm

The optimal movement of the minimum angular jerk model can be calculated analytically. Therefore, we compared the analytical solution of the minimum angular jerk model with the solution selected by the RCGA algorithm developed in this study.

The minimum hand-jerk model

The optimal solutions of the minimum hand jerk model are found analytically. Therefore, we compared the results obtained using the above algorithm (RCGA) with the optimal solution obtained analytically. As a result, the hand paths, hand trajectories and tangential velocities were in excellent agreement.

$$x(t) = x(0) + (x(0) - x(t_f))\left(15\phi^4 - 6\phi^5 - 10\phi^3\right), \quad (1)$$

$$y(t) = y(0) + (y(0) - y(t_f))\left(15\phi^4 - 6\phi^5 - 10\phi^3\right). \quad (2)$$

where

$$\phi = \frac{t}{t_f}. \quad (3)$$

The minimum angular-jerk model

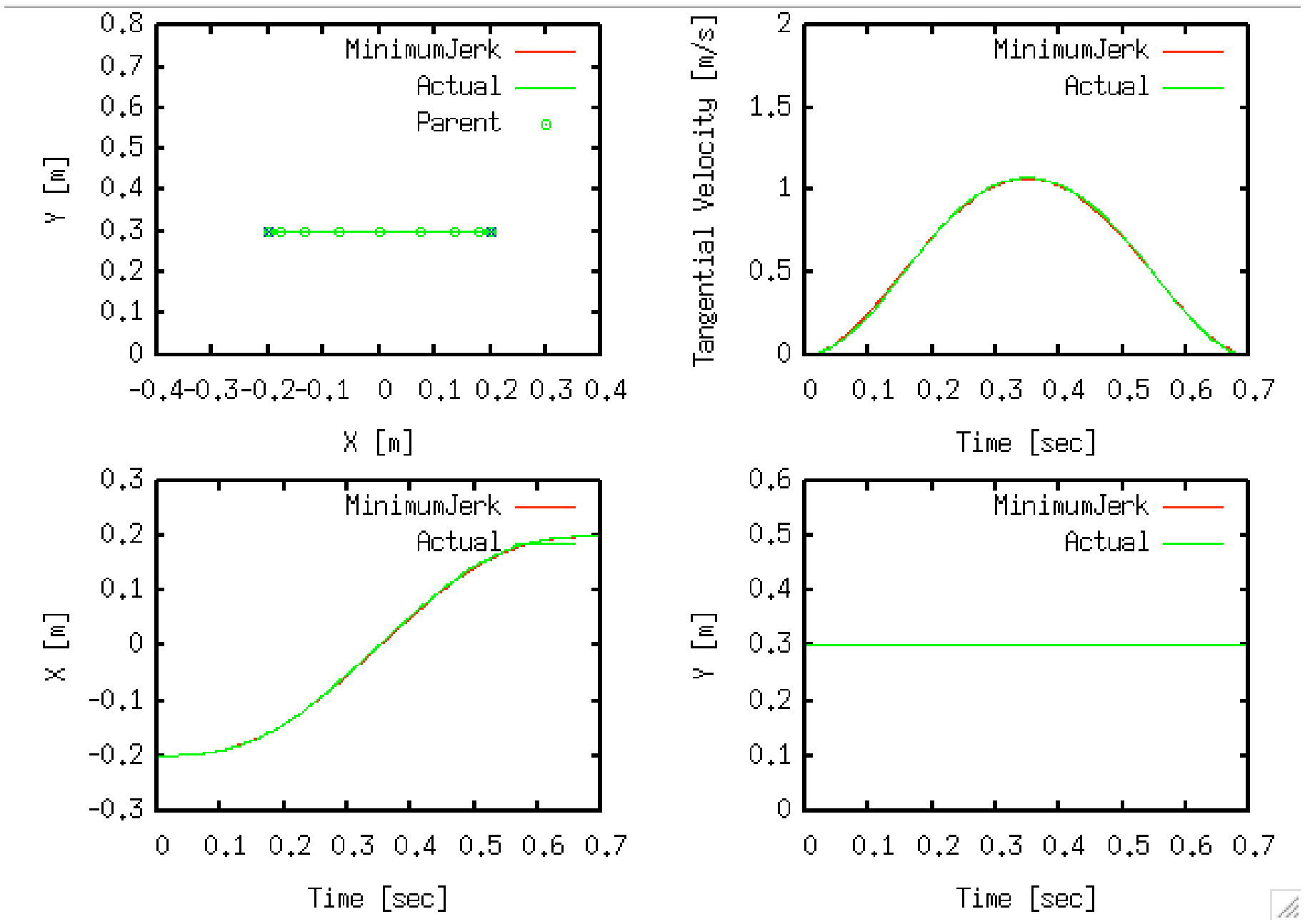

図 1 The minimum hand-jerk mode: point-to-point reaching movement (red line: analytical solution, green line: developed algorithm)

The optimal solution for the minimum angle jerk model can also be found analytically by specifying the arm posture (each joint angle) at the start and end points, in addition to the coordinates of the start and end points and the movement time. The hand trajectory can be calculated from these values. As a result, the analytical solutions of the arm postures and fingertip trajectories were also almost identical to the optimal solutions of the optimization algorithm (RCGA).

$$\theta_s(t) = \theta_s(0) + (\theta_s(0) - \theta_s(t_f)) \left(15\phi^4 - 6\phi^5 - 10\phi^3\right), \tag{4}$$

$$\theta_e(t) = \theta_e(0) + (\theta_e(0) - \theta_e(t_f)) \left(15\phi^4 - 6\phi^5 - 10\phi^3\right), \tag{5}$$

$$\theta_w(t) = \theta_w(0) + (\theta_w(0) - \theta_w(t_f)) \left(15\phi^4 - 6\phi^5 - 10\phi^3\right). \tag{6}$$

where

$$\phi = \frac{t}{t_f}. \tag{7}$$

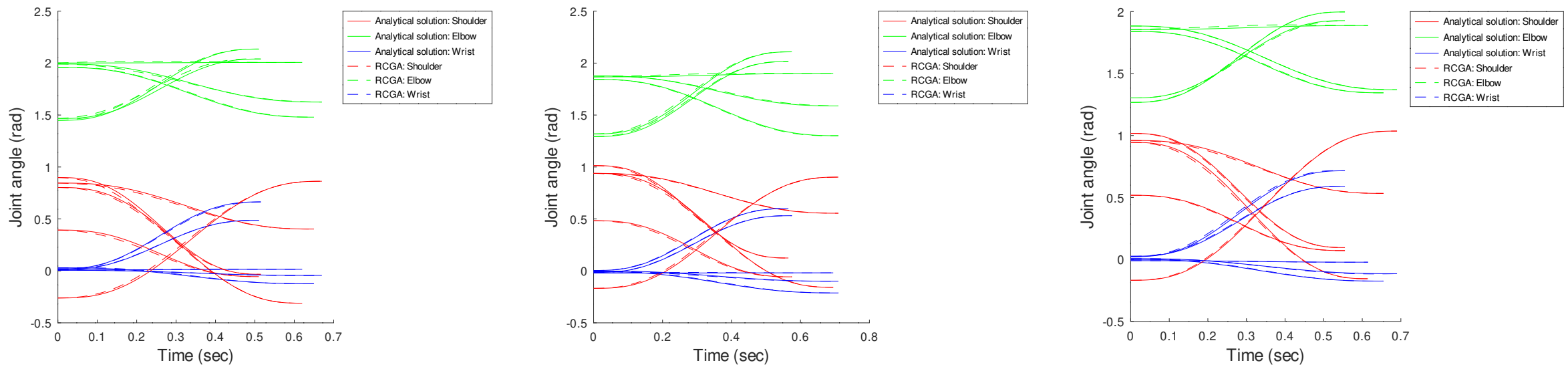

図 2 The minimum angular-jerk mode: joint angles (solid line: analytical solution, dashed line: developed algorithm. red: shoulder, green: elbow, blue: wrist).

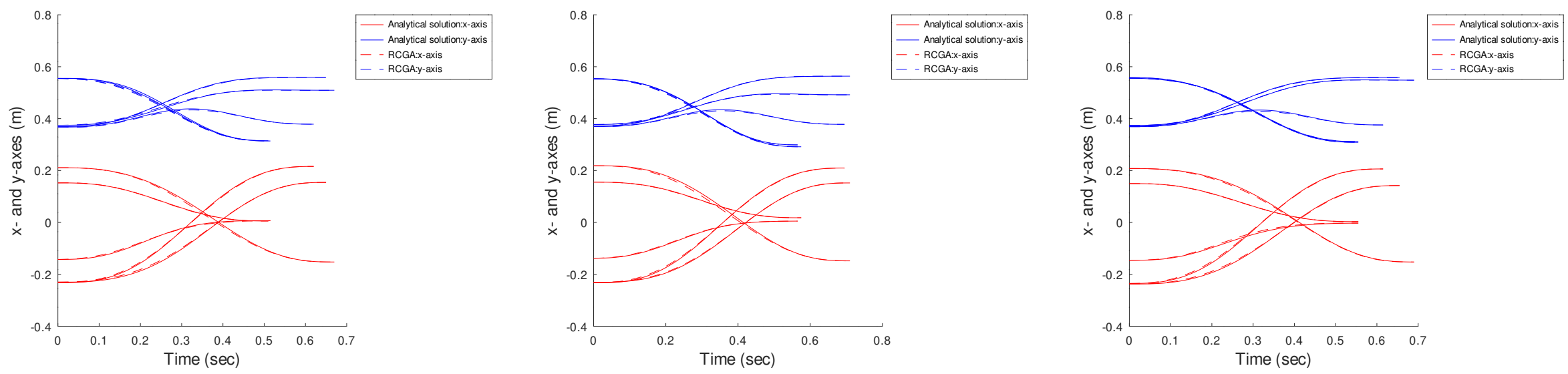

図 3 The minimum angular-jerk mode: x- and y-axes (solid line: analytical solution, dashed line: developed algorithm, red: x-axis, blue: y-axis).

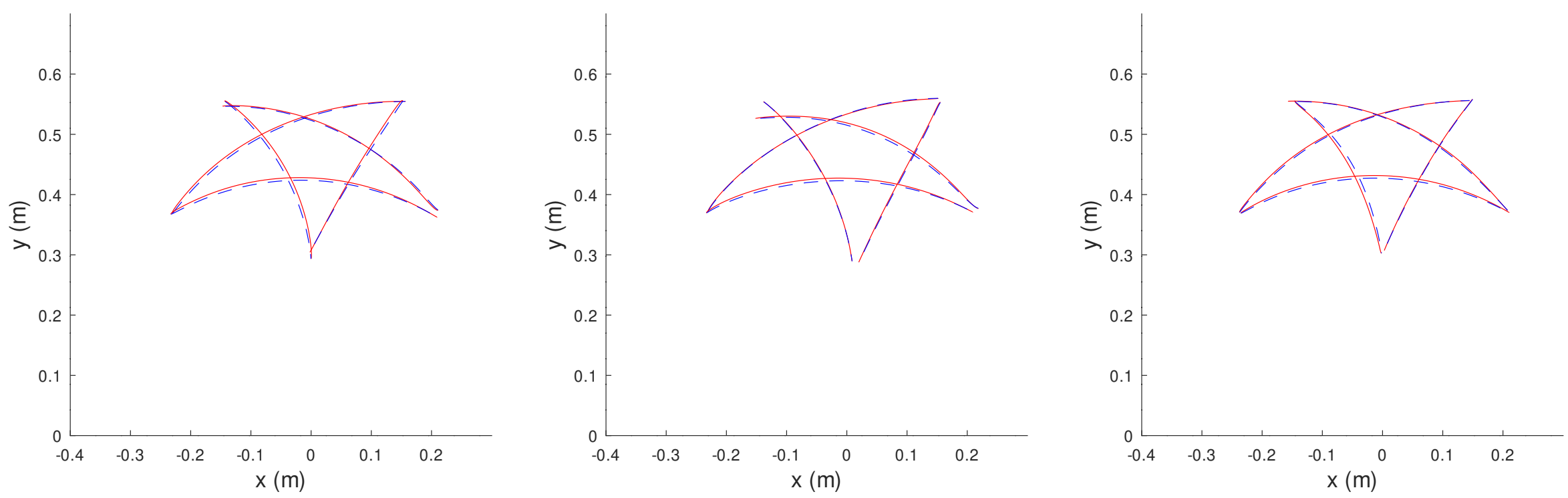

図 4 The minimum angular-jerk mode: fingertip paths (solid line: analytical solution, dashed line: developed algorithm).
