# Supplementary material for: Muscle-level evaluation of the minimum muscle-stress-change model in human three-joint reaching using anatomically expanded arm models
Source: Biol Cybern. 2026 Aug 22;120(5-6):29. doi: 10.1007/s00422-026-01056-2 (PMC13499790; doi:10.1007/s00422-026-01056-2)
Supplement: Supplementary file 2 [file 422_2026_1056_MOESM2_ESM.pdf]

Dynamics equation for a three-joint arm

Katayama, Masazumi
Division of Human and Artificial Intelligent Systems,
Graduate School of Engineering, University of Fukui, Japan

The dynamics equation for a three-joint arm in a horizontal plane is described as

$$\mathbf{M}\ddot{\boldsymbol{\theta}} + \mathbf{H} + \mathbf{D}\dot{\boldsymbol{\theta}} = \boldsymbol{\tau}, \tag{1}$$

where a joint torque vector $\boldsymbol{\tau}$, an angular velocity vector $\dot{\boldsymbol{\theta}}$ and an angular acceleration vector $\ddot{\boldsymbol{\theta}}$ denote

$$\boldsymbol{\theta} = \begin{pmatrix} \theta_1 \\ \theta_2 \\ \theta_3 \end{pmatrix}, \ \dot{\boldsymbol{\theta}} = \begin{pmatrix} \dot{\theta}_1 \\ \dot{\theta}_2 \\ \dot{\theta}_3 \end{pmatrix}, \ \ddot{\boldsymbol{\theta}} = \begin{pmatrix} \ddot{\theta}_1 \\ \ddot{\theta}_2 \\ \ddot{\theta}_3 \end{pmatrix}, \ \boldsymbol{\tau} = \begin{pmatrix} \tau_1 \\ \tau_2 \\ \tau_3 \end{pmatrix},$$

where the subscript i of θ_i and τ_i represents the i-th joint: The shoulder is 1, the elbow is 2 and the wrist is 3. Inertia matrix $\mathbf{M}$ and Coriolis and centrifugal force vector $\mathbf{H}$ are expressed as

$$\mathbf{M} = \begin{pmatrix} M_{11} & M_{12} & M_{13} \\ M_{21} & M_{22} & M_{23} \\ M_{31} & M_{32} & M_{33} \end{pmatrix}, \ \mathbf{H} = \begin{pmatrix} H_1 \\ H_2 \\ H_3 \end{pmatrix}.$$

$$
\begin{aligned}
M_{11} &= \sigma_1 + \sigma_2 + \sigma_3 + 2b_1 + 2b_2 + 2b_3 \\
M_{12} &= \sigma_2 + \sigma_3 + b_1 + b_2 + 2b_3 \\
M_{13} &= \sigma_3 + b_2 + b_3 \\
M_{21} &= \sigma_2 + \sigma_3 + b_1 + b_2 + 2b_3 \\
M_{22} &= \sigma_2 + \sigma_3 + 2b_3 \quad M_{23} = \sigma_3 + b_3 \\
M_{31} &= \sigma_3 + b_2 + b_3 \quad M_{32} = \sigma_3 + b_3 \quad M_{33} = \sigma_3 \\
H_1 &= -(b_4 + b_5)(2\dot{\theta}_1 + \dot{\theta}_2)\dot{\theta}_2 \\
&\qquad -(b_5 + b_6)(2\dot{\theta}_1 + 2\dot{\theta}_2 + \dot{\theta}_3)\dot{\theta}_3 \\
H_2 &= (b_4 + b_5)\dot{\theta}_1{}^2 - b_6(2\dot{\theta}_1 + 2\dot{\theta}_2 + \dot{\theta}_3)\dot{\theta}_3 \\
H_3 &= (b_5 + b_6)\dot{\theta}_1{}^2 + b_6(2\dot{\theta}_1 + \dot{\theta}_2)\dot{\theta}_2 \\
b_1 &= C_2\sigma_4 L_1 \quad b_2 = C_{23}\sigma_6 L_1 \quad b_3 = C_3\sigma_6 L_2 \\
b_4 &= S_2\sigma_4 L_1 \quad b_5 = S_{23}\sigma_6 L_1 \quad b_6 = S_3\sigma_6 L_2 \\
\sigma_1 &= I_1 + (m_2 + m_3)L_1{}^2 \quad \sigma_2 = I_2 + m_3 L_2{}^2 \\
\sigma_3 &= I_3 \quad \sigma_4 = m_2 L_{g2} + m_3 L_2 \quad \sigma_6 = m_3 L_{g3} \\
C_2 &= \cos(\theta_2) \quad C_3 = \cos(\theta_3) \quad C_{23} = \cos(\theta_2 + \theta_3) \\
S_2 &= \sin(\theta_2) \quad S_3 = \sin(\theta_3) \quad S_{23} = \sin(\theta_2 + \theta_3)
\end{aligned}
$$

Here, L_i, L_{gi} and m_i are the link length, center of mass and mass of the ith link, and I_i is the moment of inertia around the ith joint. Links 1, 2, and 3 correspond to the upper arm, forearm, and hand, respectively
