# Supplementary material for: Muscle-level evaluation of the minimum muscle-stress-change model in human three-joint reaching using anatomically expanded arm models
Source: Biol Cybern. 2026 Aug 22;120(5-6):29. doi: 10.1007/s00422-026-01056-2 (PMC13499790; doi:10.1007/s00422-026-01056-2)
Supplement: Supplementary file 3 [file 422_2026_1056_MOESM3_ESM.pdf]

Supplementary results

Katayama, Masazumi

Division of Human and Artificial Intelligent Systems,

Graduate School of Engineering, University of Fukui, Japan

1 Sensitivity to PCSA

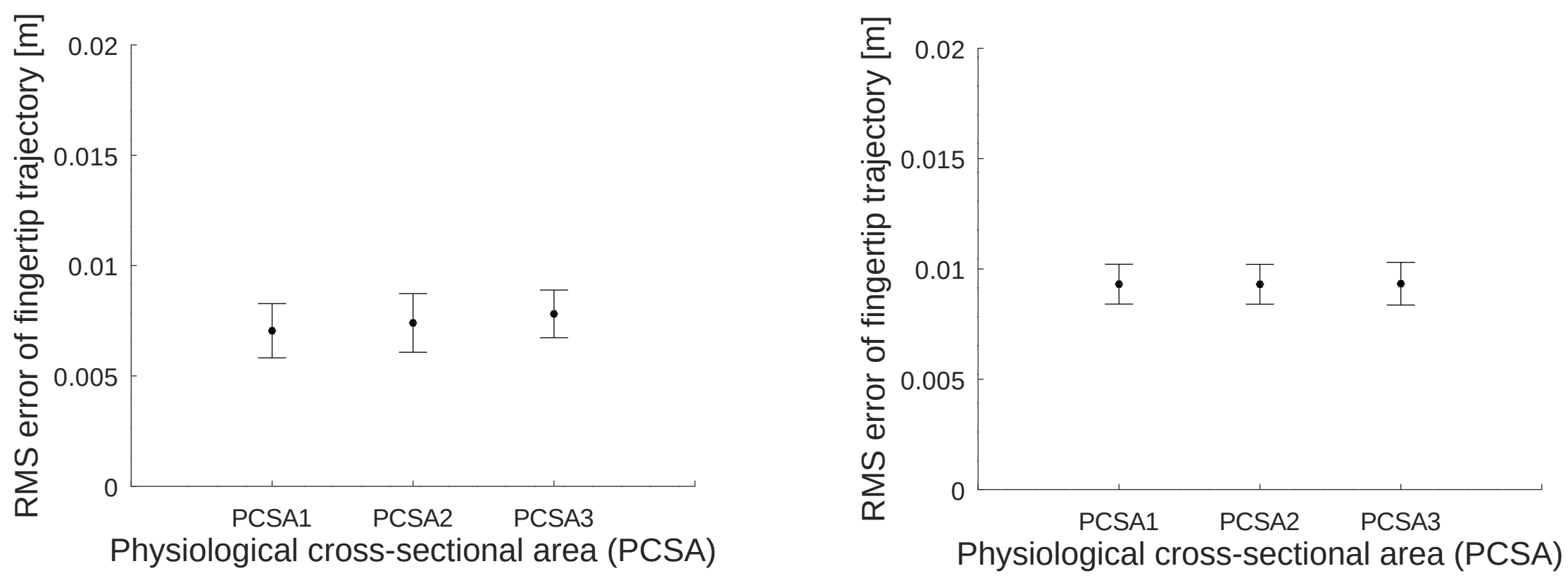

a MSC_2 **b** MTC

Fig.1 RMS errors of the optimal fingertip trajectories. (Muscle selection: S22)

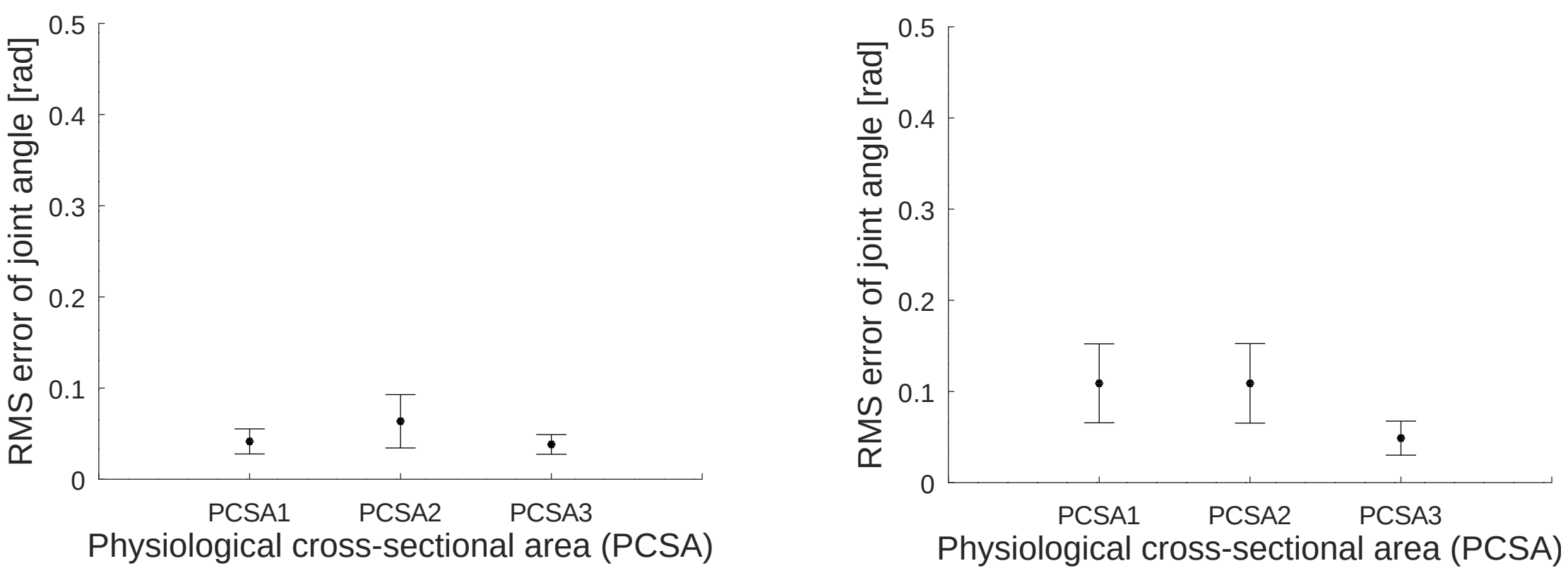

a MSC_2 **b** MTC

Fig.2 RMS errors of the arm postures. (Muscle selection: S22)

2 Muscle selection

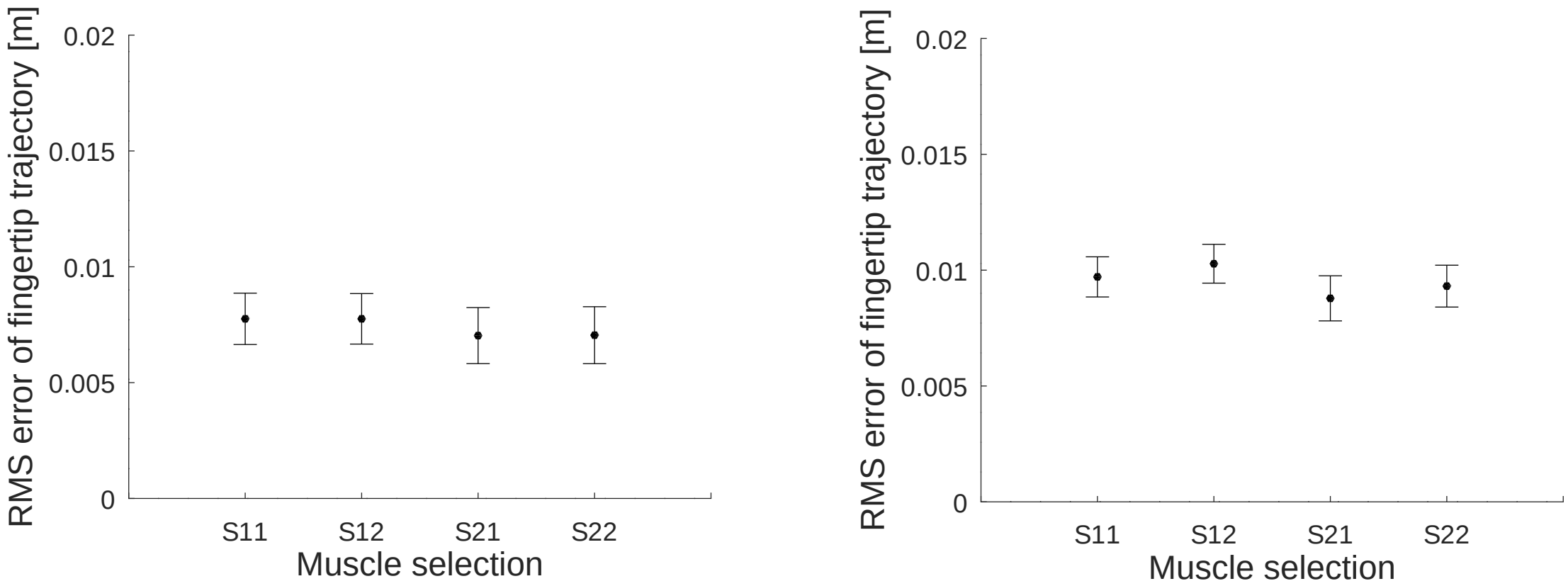

a MSC_2 **b** MTC

Fig.3 RMS errors of the optimal fingertip trajectories. (PCSA: PCSA1)

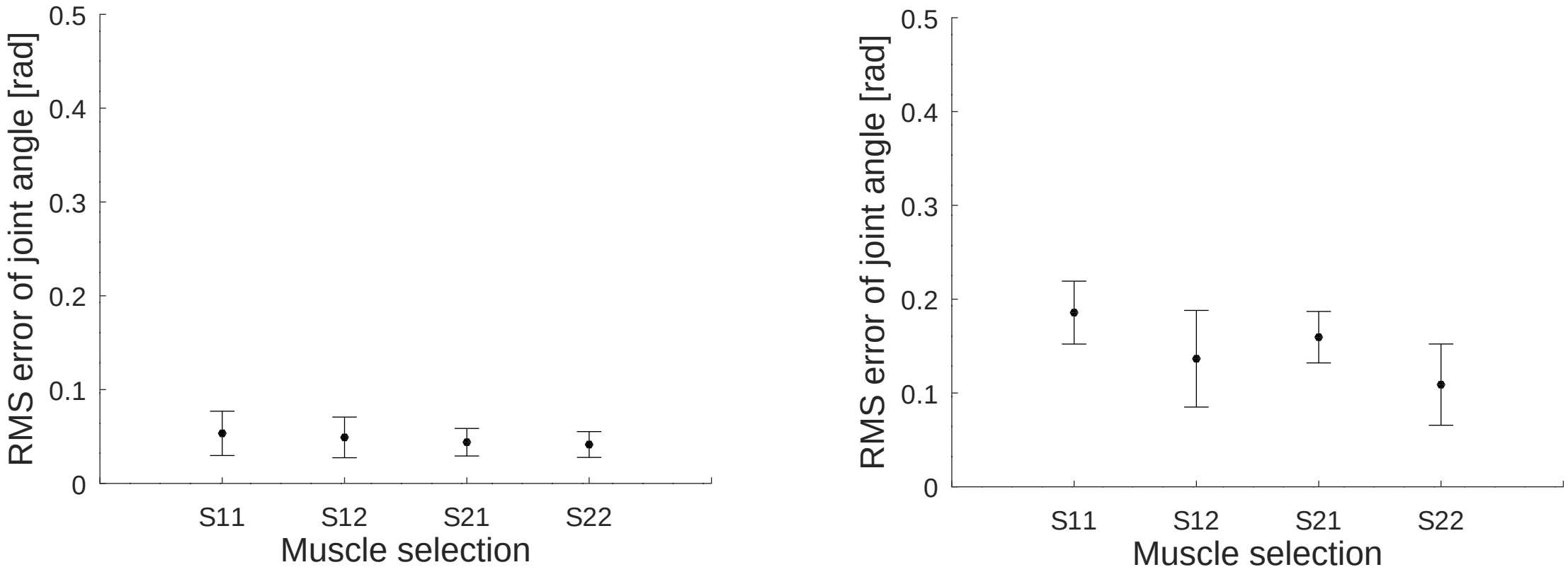

a MSC_2 **b** MTC

Fig.4 RMS errors of the arm postures. (PCSA: PCSA1)

3 Viscosity dependence

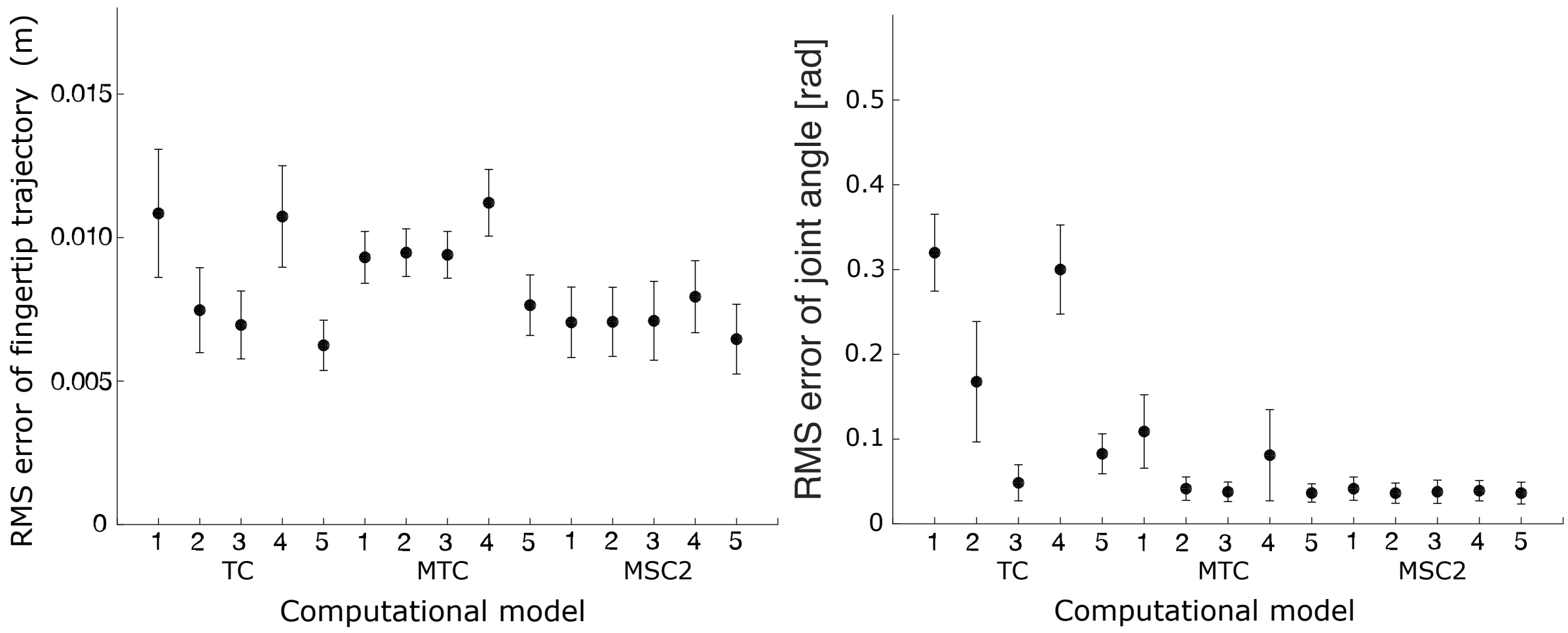

a Fingertip trajectory **b** Arm posture

Fig.5 RMS errors of the optimal fingertip trajectories and arm postures selected by the TC, MTC and MSC_2 models. 1: The viscous conditions in this study, 2: the condition where only the wrist joint is doubled, 3: the condition where only the wrist joint is quadrupled, 4: the condition where the shoulder and elbow joints are halved, and 5: the condition where the wrist joint is quadrupled and the shoulder and elbow joints are doubled. (Muscle selection: S22. PCSA: PCSA1)
